# ESE-PWDNet: an efficient early-stage pine wilt disease detection network

**DOI:** 10.3389/fpls.2026.1841429

**Published:** 2026-06-08

**Authors:** Zhemin Ma, Fang Wang, Haifeng Lin

**Affiliations:** 1 College of Information Science and Technology, Nanjing Forestry University, Nanjing, China; 2 College of Electronic Engineering, Nanjing XiaoZhuang University, Nanjing, China

**Keywords:** computer vision, deep learning, pine wilt disease, small object detection, UAV imagery

## Abstract

To address the challenge of difficult small target recognition in the early detection of Pine Wilt Disease (PWD), this study proposes an efficient Unmanned Aerial Vehicle remote sensing detection model named ESE-PWDNet (Efficient Small-scale Early PWD Detection Network). Using a DJI Air3 UAV platform, a multi-temporal and multi-view high-resolution dataset of early-stage PWD was independently constructed in the Tangshan Forest Area, Jiangning District, Nanjing City, Jiangsu Province. Based on this dataset, a key module—the Efficient Visual Linear Unit (EFVLU)—was designed. Serving as the foundational building block of ESE-PWDNet, the EFVLU combines with convolutional modules (Conv) to form the backbone network, which efficiently captures global dependencies and improves the detection of small targets through global context while reducing computational complexity. Furthermore, inspired by the PANet architecture and utilizing the Attention State Space Block (ASSB), a novel neck network was designed to empower the model with efficient high-resolution image processing capabilities while maintaining high computational efficiency. In the prediction head, the introduction of the Efficient Multi-scale Attention (EMA) mechanism and the Lightweight Shared Detail Enhanced Convolutional Detection Head (LSDECD) comprehensively enhances the model’s perception and localization capabilities for small targets with almost no additional inference computational cost. Experiments on the constructed multi-environment early PWD dataset demonstrate that ESE-PWDNet significantly improves the recognition performance of tiny disease targets in complex scenes. The final model maintains high inference efficiency, achieving a Precision (P) of 75.9% and a Recall (R) of 75.1%, with a low computational complexity of 6.5 GFLOPs and 2.6M parameters. Its comprehensive performance outperforms mainstream comparative models. This research provides a reliable technical solution and data foundation for the early and precise UAV remote sensing monitoring of forestry pests.

## 1 Introduction

Pine Wilt Disease (PWD), caused by the Pine Wood Nematode (PWN), poses a devastating threat to global forest ecosystems. Its ability to trigger rapid, widespread infestations often culminates in large-scale tree mortality within a matter of months (Yu et al., 2022). In the strategic management of PWD, identifying infected individuals at an early stage is a critical prerequisite. Timely detection enables the implementation of “precision pruning” and “immediate eradication” protocols, which are intrinsically essential for truncating the disease transmission chain (Yu et al., 2021).

Historically, forest health monitoring has predominantly relied on manual field inspections conducted by forestry personnel (Idrissi et al., 2022). However, in mountainous environments characterized by fragmented terrain and poor accessibility, this conventional approach is severely constrained by exorbitant labor and time costs. Furthermore, manual assessments are inherently susceptible to subjective variables—such as varying levels of professional expertise, visual blind spots, and physiological fatigue. These factors compromise the standardization and objectivity of the monitoring results, rendering real-time, dynamic health assessments of large-scale forests practically unfeasible (Shi et al., 2024).

Driven by advancements in aerial imaging, remote sensing has emerged as a pivotal technological paradigm for forest disease identification (Zhang et al., 2020). Satellite remote sensing, leveraging multi-spectral and multi-temporal capabilities, has demonstrated significant utility in wide-area coverage and macroscopic dynamic monitoring (Kang and Hong, 2016). Nevertheless, when applied to the precision monitoring of early-stage PWD, satellite platforms face inherent physical limitations. Their restricted spatial resolution frequently induces the “mixed pixel” effect. Furthermore, frequent cloud cover, high data acquisition costs, and prolonged revisit cycles hinder timely and precise feedback on disease dynamics (Han et al., 2019).

To overcome the spatial limitations of satellite platforms, Unmanned Aerial Vehicle remote sensing has been widely adopted. UAVs offer superior operational flexibility, cost-effectiveness, and immunity to atmospheric cloud interference, thereby providing ultra-high spatio-temporal resolution imagery (Han et al., 2019). Initial analytical approaches applied to UAV imagery heavily relied on traditional computer vision techniques, utilizing superpixel segmentation and handcrafted features for classification (Zhang et al., 2022). While functional in restricted contexts, these methods suffer from poor generalization when exposed to severe illumination fluctuations. Moreover, the cumbersome nature of manual feature engineering is computationally inadequate for the real-time processing demands of massive UAV datasets (Diez et al., 2021).

In recent years, deep learning architectures—predominantly Convolutional Neural Networks (CNNs) and Vision Transformers (ViTs)—have revolutionized feature extraction paradigms in remote sensing (Lo et al., 2024). However, as forestry monitoring shifts its focus toward the preventive detection of early-stage, minute lesions, a new critical bottleneck has emerged: achieving robust identification of extremely small targets amidst the severe interference of high canopy closure and complex lighting variations (Syifa et al., 2020).

Existing general-purpose object detection models lack the architectural mechanisms necessary to deeply mine the weak pathological features specific to early PWD. When deployed on wide-area, high-resolution UAV imagery, these conventional networks struggle to reconcile computational overhead with detection accuracy, thereby preventing low-latency deployment on resource-constrained forestry edge devices (Liu et al., 2020; Fouda et al., 2022). To overcome the limitations of standard architectures, there is an urgent need for a paradigm shift. Specifically, a novel network architecture is required that leverages linear-complexity operators (such as State Space Models) (Gu and Dao, 2023; Dao and Gu, 2024) to achieve expansive global perception, while concurrently deploying fine-grained representation strategies to precisely localize tiny lesions (Pei et al., 2024). This dual-capability framework would secure high-speed inference while drastically elevating the detection accuracy of early-stage PWD.

In response to the severe algorithmic bottlenecks posed by early-stage PWD small-target detection against complex forest backgrounds, we propose a novel lightweight detection model, the ESE-PWDNet. This framework elegantly balances high-precision disease identification with low computational complexity, avoiding the traditional trade-off between accuracy and efficiency. The main contributions of this work are systematically summarized as follows:

Dataset Construction: Based on high-resolution UAV data acquisition across diverse ecological regions—specifically, the Tangshan Forest Area (Nanjing) and Xinbin Manchu Autonomous County (Fushun)—a high-quality PWD dataset comprising 2,775 images was meticulously established. This dataset comprehensively covers various infection stages, with a specific emphasis on characterizing early-stage (*PWD-pre*) minute lesions.

Architectural Innovation: We innovatively designed the lightweight ESE-PWDNet architecture, systematically reconstructing the backbone, neck, and detection head to overcome small-target detection bottlenecks:

- A Novel EF-Backbone: We designed a specific feature enhancement module, the EFVLU, which significantly amplifies fine-grained detail extraction for minute lesions. Leveraging this module, the proposed EF-Backbone ensures highly robust perceptual capacity and stable localization precision across varying UAV flight altitudes.
- An Optimized Neck Structure: Inspired by the CSPNet architecture, we integrated the ASSB to construct a new feature fusion neck. This design effectively balances hierarchical feature capture and global semantic awareness, maintaining high feature discriminability without incurring heavy computational overhead.
- An Advanced Detection Head: We engineered a highly efficient detection head by introducing the LSDECD to mathematically reduce parameter redundancy, the EMA mechanism to seamlessly suppress complex forest background interference via cross-dimensional interactions, and the composite Focaler-MPDIoU loss function to provide rigorous geometric constraints for optimal early-stage bounding box regression.

Comprehensive Evaluation: Extensive evaluations on the self-constructed dataset, supported by rigorous ablation studies, quantitatively validate the incremental contributions of each structural component. Furthermore, targeted stress tests confirm the model’s exceptional robustness and cross-regional generalization capabilities across varying stand structures (homogeneous vs. heterogeneous), flight altitudes, and anthropogenic interferences.

Practical Deployment Value: This study delivers a highly efficient, lightweight, and reliable algorithmic solution specifically optimized for resource-constrained edge computing environments (e.g., UAV-mounted platforms). By enabling real-time, early-stage monitoring and precise intervention, the proposed approach can effectively curb the spread of the epidemic during its critical phase, thereby safeguarding pine forest ecological security and supporting dynamic forestry management.

## 2 Materials and methods

### 2.1 Overall research scheme

To address the core bottlenecks of early-stage Pine Wilt Disease (PWD) detection in complex forest environments—specifically the extreme small scale of lesions and the difficulty of feature identification (Liu et al., 2020)—this study first leveraged a low-altitude UAV remote sensing platform. Through image acquisition and precise annotation across diverse environmental conditions, a high-resolution specialized dataset for early-stage PWD was systematically constructed, providing robust data support for model training and performance evaluation.

On this basis, an efficient deep learning network model optimized for small object detection, ESE-PWDNet, was developed. By integrating EFVLU and Attentive State Space mechanisms (Guo et al., 2024), the model aims to achieve deep mining of weak pathological features while significantly optimizing parameter counts and computational efficiency (Gu and Dao, 2023; Pei et al., 2024). This architecture effectively resolves the inherent conflict between detection accuracy and inference speed encountered by traditional models when processing large-scale, high-resolution forestry imagery.

**Figure 1:**
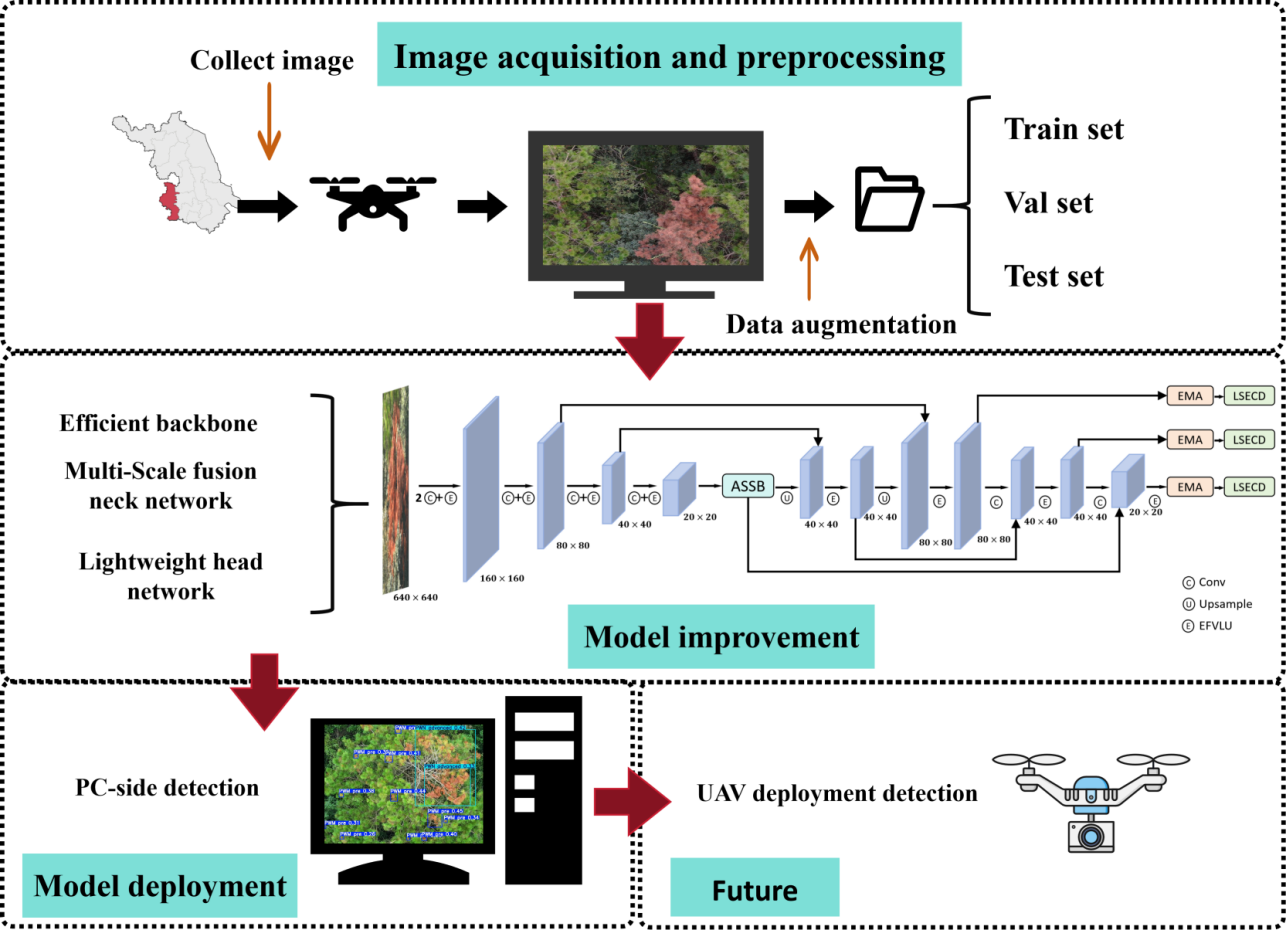
The overall research route.

Furthermore, beyond algorithmic performance enhancement, this study explores the deployment feasibility and application prospects of ESE-PWDNet on embedded forestry monitoring platforms (Fouda et al., 2022). The objective is to provide a closed-loop technical solution for regional real-time monitoring and rapid early warning of PWD. The overall logical framework and core technical route of this research are illustrated in **Figure 1**.

### 2.2 Study area and data acquisition

**Figure 2:**
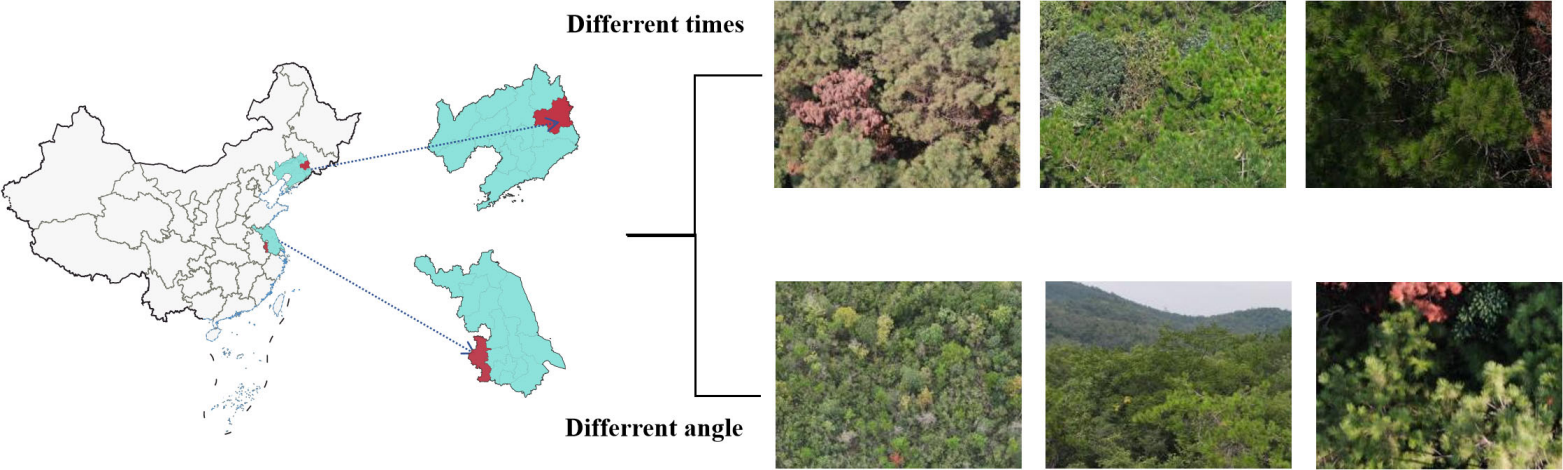
Location and construction of the dataset.

Jiangsu Province is situated along the eastern coast of China, occupying the lower reaches of the Yangtze River and the shores of the Yellow Sea. The topography gently inclines from the southwest to the northeast, dominated by expansive plains interspersed with undulating hills and low mountains. The provincial forest coverage rate is approximately 24.03%, with coniferous forests accounting for 26.17% of the total forested area. Forestry resources are primarily concentrated in the Southern Jiangsu hills, the Ningzhen Mountains, and the riparian zones of the Taihu Lake Basin (**Figure 2**).

The primary southern research site is located at Jiangjun Mountain, Nanjing (31°55′ N, 118°45′ E), situated at the junction of the Yuhuatai and Jiangning Districts. As an extension of the western Ningzhen Mountains adjacent to the Niushoushan Scenic Area, Jiangjun Mountain features a terrain of low hills with gentle slopes and crisscrossing ravines. It lies within a typical northern subtropical monsoon climate zone, characterized by a complex ecosystem of interlaced forests, hills, and water bodies. The vegetation is well-preserved, with a predominance of mixed coniferous and broad-leaved forests. Common species include *Pinus massoniana*, *Pinus thunbergii*, *Liquidambar formosana*, and *Quercus* spp., featuring a concentrated distribution of pine stands. Due to its peri-urban location and frequent anthropogenic activities, Jiangjun Mountain faces persistent threats from the invasion and dispersal of PWD (Osco et al., 2021). The sporadic distribution and rapid spreading trend of the epidemic make this a highly representative area for investigating the mechanisms and monitoring technologies of peri-urban forest pests (Diez et al., 2021).

Liaoning Province is located in the southern part of Northeast China, bounded by the Yellow Sea and the Bohai Sea to the south. The topography slopes from the northeast to the southwest, consisting mainly of plains and hills, with mountains in the east and low hills in the west. The province maintains a forest coverage rate of approximately 42.5%, with coniferous forests representing about 26.3% of the forest resources.

The primary northern research site is located in Xinbin Manchu Autonomous County, Fushun City (41°14′–41°58′ N, 124°15′–125°27′ E), situated in eastern Liaoning within the extension of the Changbai Mountains. Serving as a critical water conservation area and ecological barrier in eastern Liaodong, the geomorphology is characterized by mid-to-low mountains and hills with a typical temperate continental monsoon climate. The region is exceptionally rich in forest resources (coverage exceeding 70%), dominated by secondary forests and plantations. Vegetation types primarily consist of mixed coniferous-broadleaved forests featuring species such as *Pinus koraiensis*, *Larix* spp., and *Quercus mongolica*. Situated at the intersection of the Changbai and North China floras, and influenced by historical logging, Xinbin faces long-term risks of PWD invasion (Yu et al., 2022). This makes it a quintessential region for researching forest ecosystem stability and integrated pest management in the mountainous areas of Northeast China (Fouda et al., 2022).

The dataset collection for this study was concluded on March 1, 2025, utilizing a DJI Air3 UAV (SZ DJI Technology, Shenzhen, China). The aircraft is integrated with the Advanced Pilot Assistance System (APAS 5.0), facilitating omnidirectional obstacle avoidance, precise hovering, and automated return-to-home functionalities, which are crucial for safe navigation in complex forest canopies (Han et al., 2019). High-resolution RGB imagery was captured using the onboard 1/1.3-inch CMOS wide-angle camera. During the missions, the UAV maintained a constant flight speed with an 85% forward and lateral overlap to ensure comprehensive scanning coverage and minimize spatial blind spots, triggering the shutter every 2–3 seconds (Zhang et al., 2022). Specifically, data acquisition was scheduled four times daily at 3-hour intervals from 09:00 to 18:00. This collection cycle spanned five consecutive days to capture the progression of PWD infection across different temporal windows, ensuring the dataset’s broad temporal representativeness.

To enhance dataset diversity and the model’s robust generalization in mixed forest environments, a wide array of heterogeneous samples was intentionally incorporated (Liu et al., 2020). First, the acquisition environment featured complex backgrounds, including significant proportions of bare soil, snags (dead trees), fallen branches, and various broad-leaved species, simulating real-world noise. Second, imagery was collected across three distinct diurnal periods (morning, noon, and evening) to encompass varying illumination conditions (standard, intense, and low light), thereby mitigating the model’s sensitivity to shadow interference (Syifa et al., 2020). Furthermore, data were captured from multi-angle perspectives (low-angle, high-angle, and nadir views) at different altitudes. The finalized dataset comprises pine samples across a continuous spectrum of health statuses, ranging from healthy individuals to those in various stages of PWD infection, providing a rigorous data foundation for early-stage lesion detection.

### 2.3 Dataset construction and preprocessing

**Figure 3:**
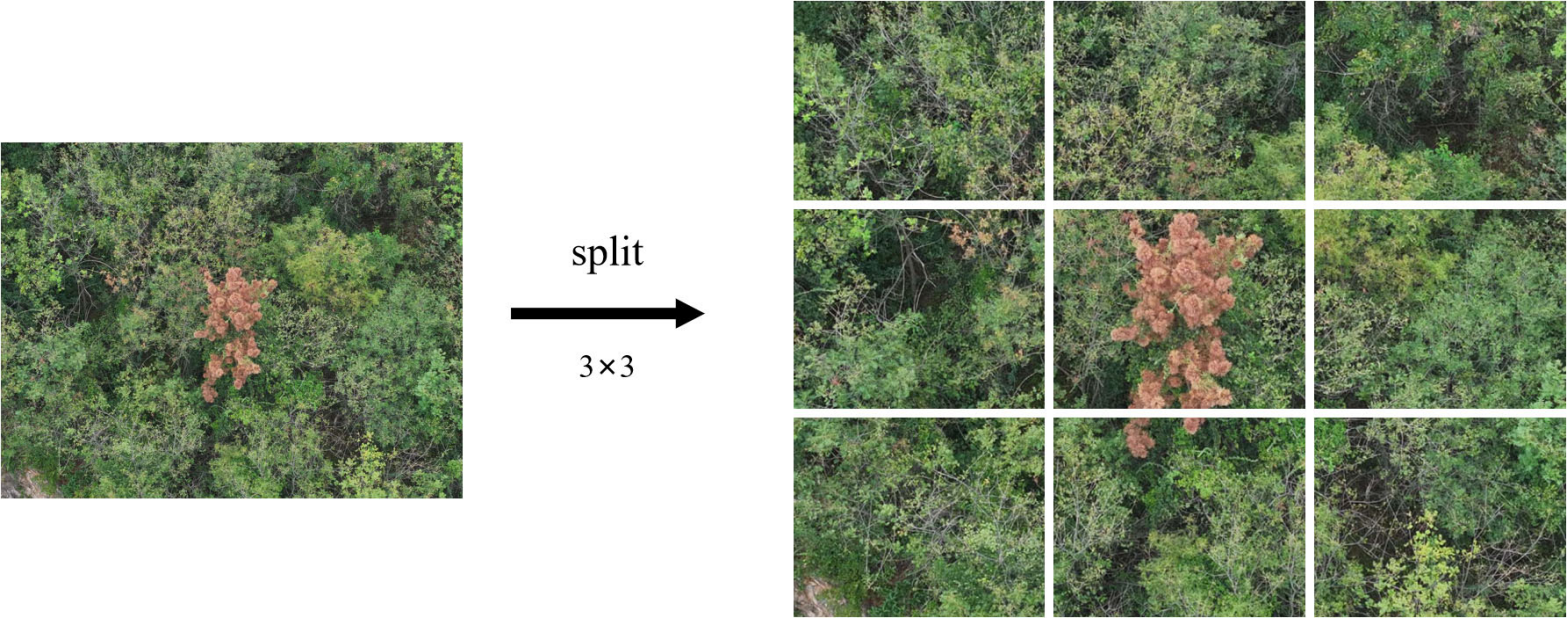
Image segmentation.

To enhance data processing efficiency and minimize computational resource consumption, the original high-resolution images were uniformly partitioned into a 3×3 grid. Each resultant tile has a resolution of 1333 × 1000 pixels, which effectively preserves essential pathological details while significantly reducing the data volume per input. This tiling strategy substantially alleviates the computational overhead during model training. Sample images after the partitioning process are illustrated in **Figure 3**.

**Figure 4:**
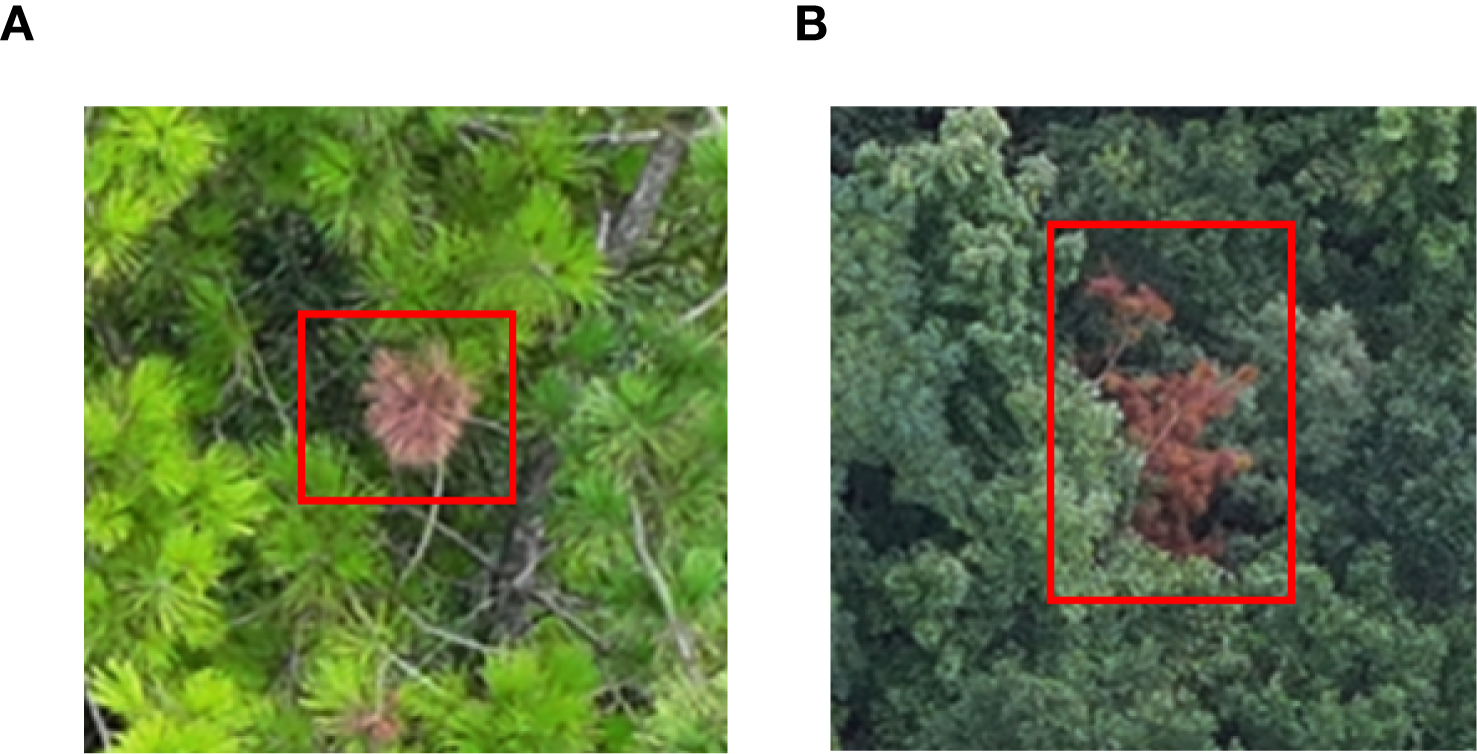
The two critical progression stages of PWD. **(A)** PWD-pre. **(B)** PWD-advanced.

Based on the clinical pathological manifestations and their corresponding visual characteristics in UAV RGB imagery (Syifa et al., 2020), this study categorizes the progression of PWD into two critical stages: the early stage (*PWD-pre*) and the advanced stage (*PWD-advanced*), as illustrated in **Figure 4**.

Early stage (*PWD-pre*): This corresponds to the initial phase of nematode infection, primarily characterized by localized chlorosis or sporadic yellowing of needles within the crown. In high-altitude UAV-captured imagery, these lesions typically exhibit extreme small-target characteristics, featuring subtle color variations and minimal spatial occupancy, which poses significant identification challenges (Liu et al., 2020). Critically, during this stage, the host tree has not yet completely lost its physiological functions. Timely detection and intervention, such as the targeted trunk injection of nematicides, offer a viable possibility for the infected trees to recover and be successfully salvaged (Osco et al., 2021).

Advanced stage (*PWD-advanced*): This stage encompasses the intermediate, late, and terminal (dead) phases of the disease. The needles exhibit large-scale desiccation, with colors shifting from yellowish-brown to deep brown or definitively gray (Yu et al., 2022). At this point, the tree has entirely lost its vitality, presenting distinct tonal and textural contrasts against the healthy forest background in remote sensing images, rendering them relatively easier to detect (Diez et al., 2021).

To ensure high data quality, the raw UAV imagery underwent rigorous screening to eliminate blurred or redundant frames. Following meticulous manual verification and annotation by forestry experts, a final set of 2,775 effective high-resolution samples was retained. Precise annotation was performed using the standard LabelImg tool. First, lesion areas were localized using tight bounding boxes, with four-point coordinates defining the precise position and scale of each target. Subsequently, category labels were assigned based on the severity of the pathology: early-stage lesions were uniformly labeled as *PWD-pre*, while intermediate-to-late and terminal-stage trees were merged and categorized as *PWD-advanced*. Furthermore, to enhance the representativeness of the dataset and bolster the model’s robustness against complex real-world environmental variables, targeted image pre-processing was implemented. Recognizing that practical UAV flight patrols occur at various times, the acquired imagery encompassed three distinct diurnal periods—morning, noon, and evening—which inherently introduced severe illumination fluctuations, specifically weak, strong, and standard lighting conditions. By applying adaptive pre-processing techniques (such as exposure compensation and contrast normalization) to these specific lighting variants, the visual discrepancies caused by intense solar angles and deep canopy shadows were effectively mitigated. This rigorous calibration process significantly enriched the dataset’s diversity, ensuring that the model learns intrinsic, illumination-invariant pathological features rather than overfitting to specific lighting artifacts.

The primary emphasis of this research is overcoming the fundamental bottlenecks of *PWD-pre* small-target detection (Woo et al., 2018; Liu et al., 2020). Compared to advanced-stage targets with prominent macroscopic features, early-stage lesions are characterized by extremely low pixel occupancy and intense background interference (e.g., overlapping branches, shadows, and soil). Their effective and automated identification holds substantial engineering value and academic significance for achieving the “early detection and early treatment” paradigm in modern forest pest management (Syifa et al., 2020).

To optimize training effectiveness and ensure generalization, the annotated dataset of 2,775 images was partitioned into training, validation, and test sets following an 8:1:1 ratio. The training set (2,220 images) was utilized for parameter optimization and feature learning, while the validation set (277 images) served to evaluate model performance and prevent overfitting. During the partitioning process, strict data independence was maintained to ensure zero overlap between the training and validation sets, thereby guaranteeing the objectivity of the evaluation results. Furthermore, the class distribution was kept consistent across all subsets to maintain data balance, enabling the model to adequately learn features from each category. To rigorously assess the model’s generalization capability, the test set (278 images) was composed of images that were completely excluded from the initial tiling process. This independent test set was reserved exclusively for performance verification and did not participate in any training or validation phases. The specific distribution of the subsets is detailed in **Table 1**.

**Table 1:** The number of labels samples in the dataset.

Dataset split	PWD-pre	PWD-advanced
Train	6414	1744
Test	784	210
Val	815	229
Total	8013	2183

### 2.4 Overall architecture of ESE-PWDNet

The backbone of ESE-PWDNet is tasked with the core mission of extracting lesion features from high-spatial-resolution UAV imagery. In the early stage of Pine Wilt Disease (*PWD-pre*), symptoms typically manifest as minuscule, slightly yellowish canopy clusters. Compared to the vast forest background, these targets exhibit extremely low spatial occupancy and sparse semantic information. Conventional Convolutional Neural Networks (CNNs) struggle to establish long-range dependencies within complex forest backgrounds due to their limited receptive fields, frequently leading to the loss of small-target features (Liu et al., 2020).

To address this fundamental limitation, this study designs the EFVLU as the core building block of the backbone. Drawing inspiration from the linear computational framework of Efficient visual Mamba architectures (Pei et al., 2024), the core logic of EFVLU lies in leveraging the linear complexity of State Space Models (SSMs) (Gu and Dao, 2023). This endows the network with the capacity to capture global contextual dependencies without a significant increase in computational overhead. Consequently, the model can effectively “isolate” suspected pathological regions from a global canopy perspective, highlighting tiny targets by enhancing their contrast against the background. To compensate for the inherent limitations of global modeling in capturing microscopic lesion textures, the EFVLU innovatively integrates a CGLU at its tail. By introducing depthwise convolutions and soft-attention mechanisms, the CGLU strengthens the model’s perception of local, fine-grained details (Woo et al., 2018). This synergistic “global-guidance and local-refinement” design ensures that the model suppresses complex environmental noise while preserving the subtle spatial structures of early disease targets.

In the feature fusion stage, the primary challenge for the neck network is managing the computational pressure induced by high-resolution imagery, while preventing small-target features from being semantically diluted or annihilated during deep-layer transmission. In response, this study constructs a novel neck fusion architecture by integrating the ASSB (Guo et al., 2024) with the Path Aggregation Network (PANet) structure (Dai et al., 2017). The ASSB module is specifically designed to handle high-resolution remote sensing data, where maintaining large feature map resolution is crucial for accurate small object localization. Leveraging its efficient SSM-based computational mechanism, the ASSB enables deep processing on large-scale feature maps, preventing the loss of fine pathological pixels typically caused by frequent downsampling operations (Gu and Dao, 2023). Concurrently, the PANet structure is employed to facilitate the bidirectional interaction of multi-scale features (Dai et al., 2017). PANet injects strong semantic information via top-down paths while transmitting high-resolution localization cues through bottom-up paths. This bidirectional flow effectively compensates for the spatial information deficit in deep feature maps, enhancing the model’s robustness to diseased trees with drastic scale variations.

The prediction head is responsible for mapping these highly condensed, multi-scale features into final detection bounding boxes. For *PWD-pre* detection, since the targets are extremely small, slight bounding box offsets or false positives can severely degrade the overall recall rate. Furthermore, to satisfy the deployment requirements for real-time, on-site forest monitoring edge devices (Fouda et al., 2022), the model must strike an optimal balance between inference speed and detection precision.

**Figure 5:**
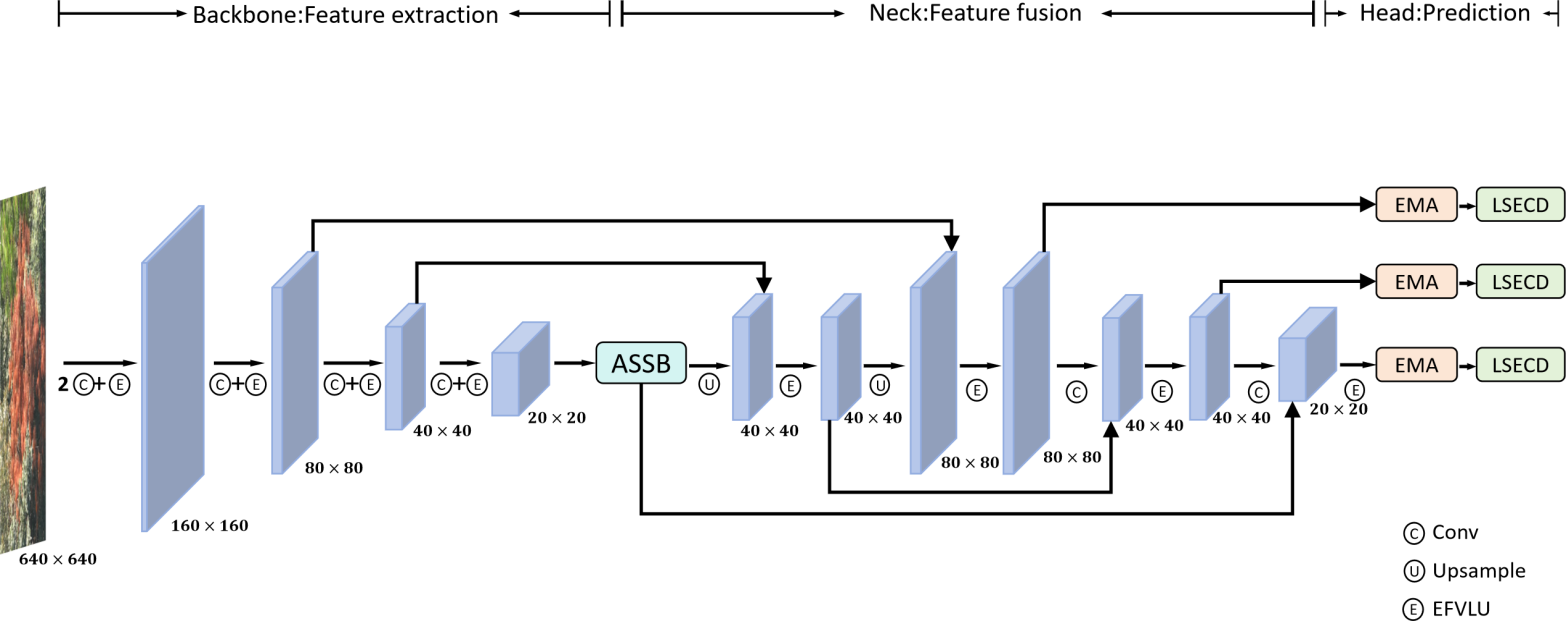
Structure of the ESE-PWDNet model.

To tackle these challenges, the prediction layer introduces the EMA mechanism and the LSDECD. The EMA module achieves fine-grained feature recalibration through cross-spatial channel interactions (Ouyang et al., 2023). It automatically focuses on frequency channels sensitive to small-target classification and suppresses cluttered light/shadow interference, making the model’s perception more acute. Furthermore, to reduce redundant computation while enhancing localization accuracy, the LSDECD head was designed. This module compresses the parameter volume through a weight-sharing mechanism and utilizes the Detail Enhanced Convolution operator to precisely delineate target boundaries. This design significantly mitigates the “localization jitter” common in small object detection, enabling the model to lock onto early infected canopies without increasing inference latency. The comprehensive architectural design of the proposed ESE-PWDNet, integrating the aforementioned modules from the backbone to the prediction head, is illustrated in **Figure 5**.

### 2.5 Backbone network

**Figure 6:**
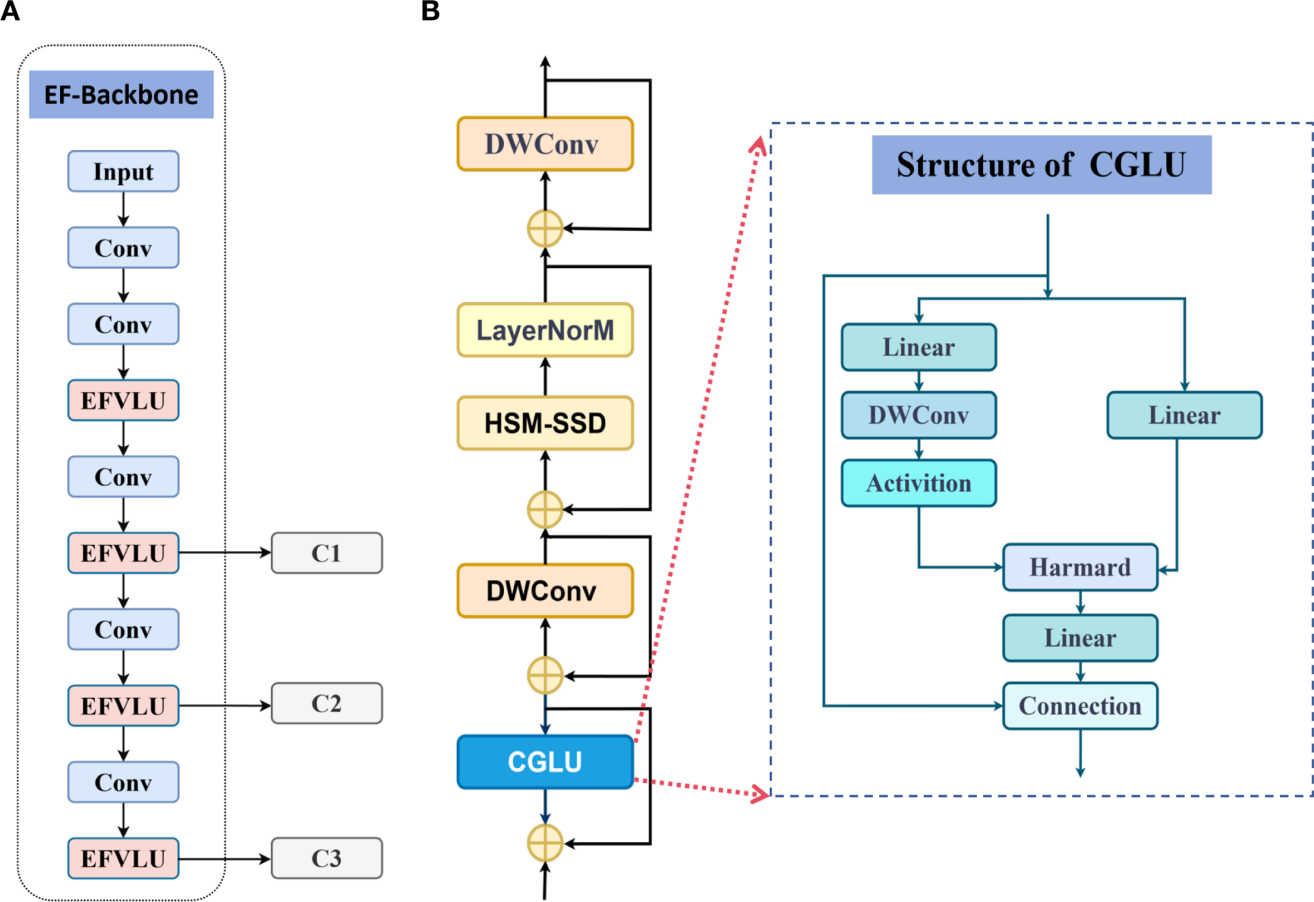
Structure of the backbone network **(a)** Structure of the EF-Backbone; **(b)** Structure of the EFVLU module.

To achieve efficient and precise capture of early-stage PWD lesions within complex, high-resolution remote sensing backgrounds, this study proposes the EF-Backbone, built upon an improved CSPNet architecture (Wang et al., 2020) (as illustrated in **Figure 6**). In UAV imagery, early-stage infected trees typically occupy only a limited number of pixels, and their spectral variations compared to surrounding healthy canopies are extremely subtle (Liu et al., 2020). CNNs are prone to severe feature loss and semantic annihilation of such minute targets during successive downsampling processes. To circumvent this bottleneck, this research designs a core building block: the EFVLU.

The architectural logic of the EFVLU module is predicated on “parallel global macro-modeling and local detail enhancement.” Its foundational framework draws inspiration from the efficient Visual Mamba blocks in EfficientVMamba (Pei et al., 2024), leveraging the linear computational complexity of SSMs (Gu and Dao, 2023) to overcome the inherent receptive field limitations encountered when processing high-resolution remote sensing data. In small object detection tasks, global contextual awareness is crucial for distinguishing genuine diseased trees from shadows and complex forest backgrounds. By establishing long-range dependencies, the EFVLU captures the macroscopic distribution patterns of forest areas, thereby significantly amplifying the model’s primary sensitivity to suspected lesion regions.

However, pure global modeling often lacks the localized inductive biases inherent in standard convolutional operations, which can result in insufficient precision when localizing the boundaries of small targets. To mitigate this, a CGLU is innovatively integrated at the terminal end of the EFVLU (Woo et al., 2018). Drawing upon the robust foveal perception principles of the TransNeXt architecture (Shi, 2024), this structure effectively compensates for the loss of spatial details by incorporating depthwise convolutional layers. The CGLU utilizes its unique gating and soft-attention mechanisms to perform secondary calibration of local spatial features. This synergistic design not only improves the feature extraction capability for microscopic lesion textures (e.g., initial needle discoloration) but also ensures highly precise localization and representation of small targets under complex lighting and occlusion. Ultimately, through the hierarchical feature extraction of the EF-Backbone, the proposed model achieves profound mining of early-stage PWD small-target features while simultaneously reducing overall computational complexity.

### 2.6 Feature fusion layer

**Figure 7:**
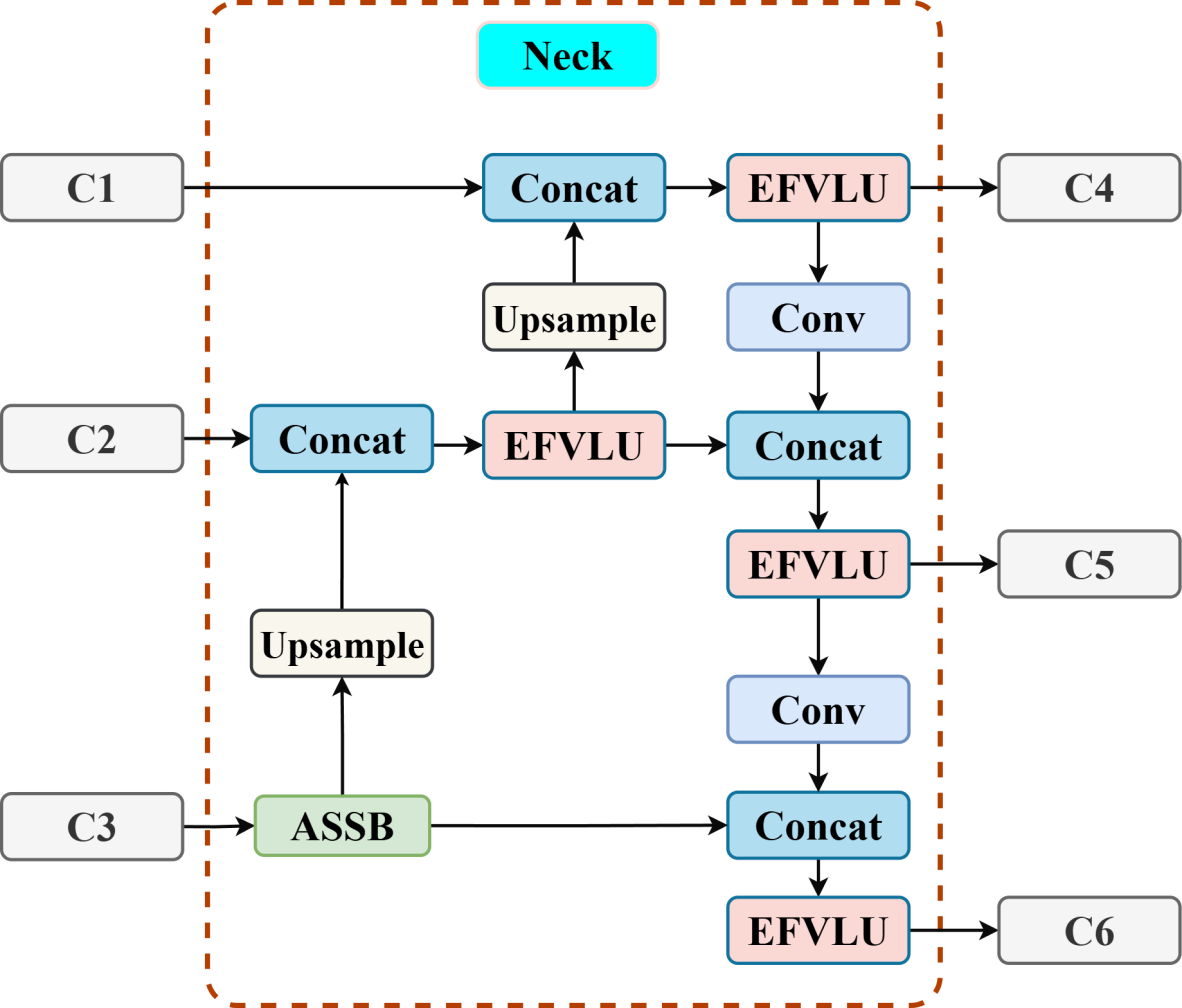
Structure of the Neck network.

**Figure 8:**
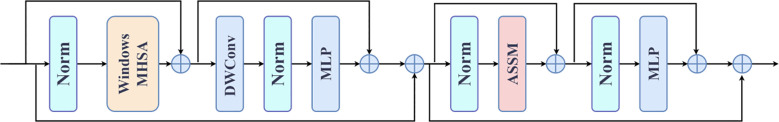
Structure of the ASSB.

Serving as the critical bridge between the EF-Backbone and the prediction head, the primary objective of the Feature Fusion Layer (Neck) is to efficiently coordinate multi-scale feature information. To address the severe challenges posed by early-stage PWD lesions in remote sensing imagery—specifically their extremely small scale, low contrast, and weak feature representation—this study constructs an enhanced feature fusion network tailored for small-target reinforcement (**Figure 7**). This architecture seamlessly integrates the PANet structure (Dai et al., 2017) with the ASSB derived from MambaIRv2 (Guo et al., 2024). The detailed topological structure of the ASSB is further illustrated in **Figure 8**.

In early-stage PWD monitoring, minute lesion targets are highly susceptible to semantic dilution and spatial information annihilation caused by successive downsampling during deep feature extraction. Consequently, the ASSB is introduced into the neck network. By deeply coupling channel attention mechanisms with the Attentive State Space Module (ASSM), this block leverages the inherent long-range dependency modeling capability of SSMs (Gu and Dao, 2023) to process high-resolution feature maps efficiently, avoiding a significant increase in computational complexity. This design ensures that the model, while fusing multi-scale features, can acutely capture the spatial distribution of subtle lesions, significantly enhancing the perceptual depth for small targets under low signal-to-noise ratio (SNR) conditions.

To further resolve the persistent issues of inaccurate localization and poor classification of small targets, the neck network adopts and optimizes the bidirectional feature aggregation architecture of PANet (Dai et al., 2017). Firstly, through the top-down Feature Pyramid Network (FPN) pathway (Lin et al., 2017), strong semantic information from deeper layers is injected into shallow feature maps via upsampling. This process endows high-resolution shallow features with robust categorical discriminative power, enabling the model to effectively distinguish suspected pathological trees from cluttered background interference. Secondly, given that small-target localization is highly dependent on spatial details, this study strengthens the Bottom-up Path Augmentation (BPA) linkage. By increasing the feature transmission paths from shallow to deep layers, the pixel distance required to convey low-level, high-resolution positional information to the top layers is substantially shortened. This bidirectional feature alignment mechanism compensates for the inherent localization deficiencies of deep networks, ensuring that the model can precisely lock onto the boundaries of early infected trees even when lesion features are severely blurred. Ultimately, the efficient perception of the ASSB and the multi-scale fusion mechanism of PANet work synergistically to construct an enhanced feature fusion architecture that optimally balances computational efficiency with small-target detection accuracy.

### 2.7 Module

#### 2.7.1 HSM-SSD

**Figure 9:**
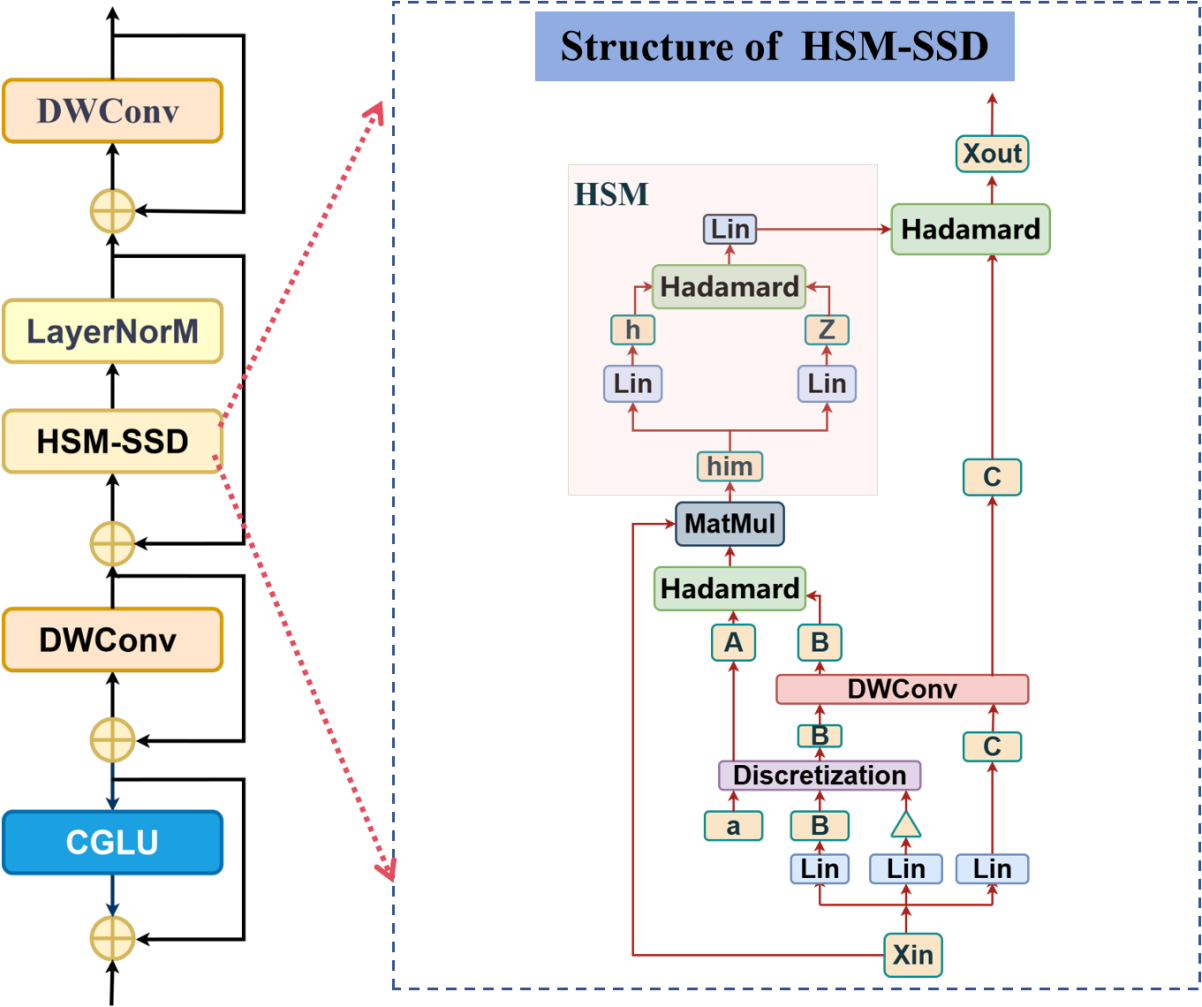
Structure of the HSM-SSD.

The HSM-SSD is a core component of the EFVLU module, with its topological structure depicted in **Figure 9**. A primary difficulty in identifying early PWD targets is their susceptibility to severe feature decay during transmission through deep network layers. The design of the HSM-SSD is fundamentally motivated by State Space Duality (SSD) (Dao and Gu, 2024), which provides a mathematical unification of the recursive and convolutional forms of linear time-invariant (LTI) systems (Gu and Dao, 2023). This dual representation allows the model to maintain long-range dependency modeling during inference while enabling convolution-like parallelization during training and feature aggregation. Consequently, it enhances the perceptual depth of global contextual information while significantly reducing computational overhead (Dao and Gu, 2024).

The core mechanism of the HSM-SSD lies in optimizing the state transition process via a latent state mixer. Its fundamental state-space equations can be expressed as Equations 1 and 2:

(1) $$\begin{array}{c} h_{t}={\bar{A}}_{t}h_{t-1}+{\bar{B}}_{t}x_{t} \end{array}$$

(2) $$\begin{array}{c} y_{t}=C_{t}h_{t} \end{array}$$

Where $x_{t}$ represents the input features, $h_{t}$ is the latent state storing critical information of small targets, $h_{t-1}$ is the latent state from the previous time step, ${\bar{A}}_{t}$ is the state transition matrix (which determines how the system evolves from $h_{t-1}$ to $h_{t}$), $\bar{B_{t}}$ is the input projection matrix, and $C_{t}$ is the observation matrix (defining how the internal latent state is mapped to the visible output $y_{t}$). To break sequential dependencies and enhance feature capture, HSM-SSD introduces a dual transformation. Expanding (Equation 1), the mapping relationship over a sequence can be represented as a matrix transformation (Equation 3):

(3) $$\begin{array}{c} y_{i}=\sum_{j=1}^{i}M_{ij}x_{j} \end{array}$$

The formula expresses the current output $y_{i}$ as a weighted sum of all historical inputs $x_{1}$ through $\;x_{i}$, where the weighting coefficients $M_{ij}$ capture how much the j-th input contributes to the i-th output. Where $M_{ij}$ is a kernel matrix with a semi-separable structure:

(4) $$\begin{array}{c} M_{ij}=C_{i}(\prod_{k=j+1}^{i}{\bar{A}}_{k}){\bar{B}}_{j}(i\geq j) \end{array}$$

(Equation 4) fundamentally models a dynamic process of spatial signal transmission and memory attenuation. To fully elucidate its mechanism in the context of early-stage PWD detection, the formula can be deconstructed into four distinct functional components: $M_{ij}$ (Contribution Weight) is the final calculated outcome, quantifying the exact degree to which the historical input feature at spatial position j influences the current state at position i.It essentially measures the “residual signal strength” after long-range transmission. ${\bar{B}}_{j\;}$(Input Signal Gain) acting as a signal amplifier at the source, this projection matrix determines the intensity of the input feature $x_{j}$ injected into the hidden state. When the model encounters a suspected microscopic lesion, it dynamically increases ${\bar{B}}_{j\;}$ to force-amplify the weak pathological signal, preventing it from being submerged by background noise. $\prod_{k=j+1}^{i}{\bar{A}}_{k}$ is Memory Decay Coefficient. As the core of the state-space mechanism, this cumulative product dictates the rate of memory attenuation across the spatial sequence. If the state transition matrix ${\bar{A}}_{k}$ is large (approaching 1), information is preserved over extended distances, establishing a “global-smooth” mode to perceive the macroscopic forest canopy. Conversely, when detecting early-stage PWD, the model adaptively decreases ${\bar{A}}_{k}$. This rapid signal decay intentionally cuts off long-range memory, triggering a “local-focus” mode that effectively filters out complex light and shadow interference from distant background regions. $C_{i}$ is Output Extractor, finally, after the initial signal is amplified by ${\bar{B}}_{j\;}$ and spatial attenuation is applied via ${\bar{A}}_{k}$, the observation matrix $C_{i}$ acts as a feature extractor. It projects the evolved, purified hidden state into the final visible output feature at the current position i.In summary, this mathematical formulation allows the HSM-SSD module to dynamically modulate its spatial attention. By actively amplifying weak lesion targets via ${\bar{B}}_{j\;}$ and simultaneously truncating irrelevant background noise via ${\bar{A}}_{k}$, the mechanism ensures that the semantic features of minute early-stage PWD lesions remain highly discriminative within complex forest environments.

The contribution of this mechanism to early PWD small-target detection is primarily reflected in its fidelity of weak semantic information and the deep dual correlation between global and local features. Addressing the problem where sparse lesion pixels are easily submerged by background noise, HSM-SSD utilizes the mixer to reorganize features in the channel dimension, increasing the latent state’s memory capacity for subtle discolored needle signals. The matrix dual mapping ensures that even if the spatial span of the target is extremely small, its key semantics can be transmitted losslessly through the global interaction link. Furthermore, this mechanism endows the model with both the local texture sensitivity of convolutional operators (for precise boundary locking) and the global consistency of state-space models (for suppressing complex forest light and shadow interference). This synergistic optimization significantly improves localization accuracy and categorical discriminability in low-SNR environments, providing robust algorithmic support for the efficient monitoring of early PWD targets.

#### 2.7.2 ASSM

**Figure 10:**
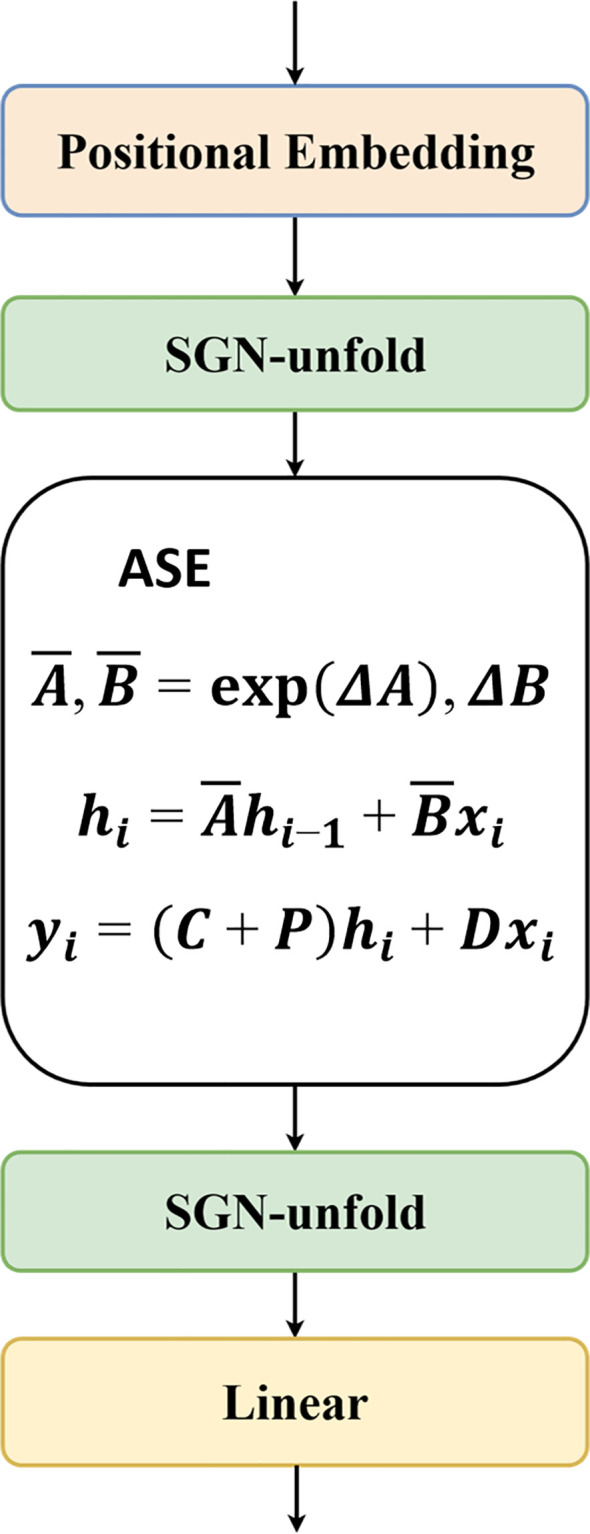
Structure of the ASSM.

**Figure 11:**
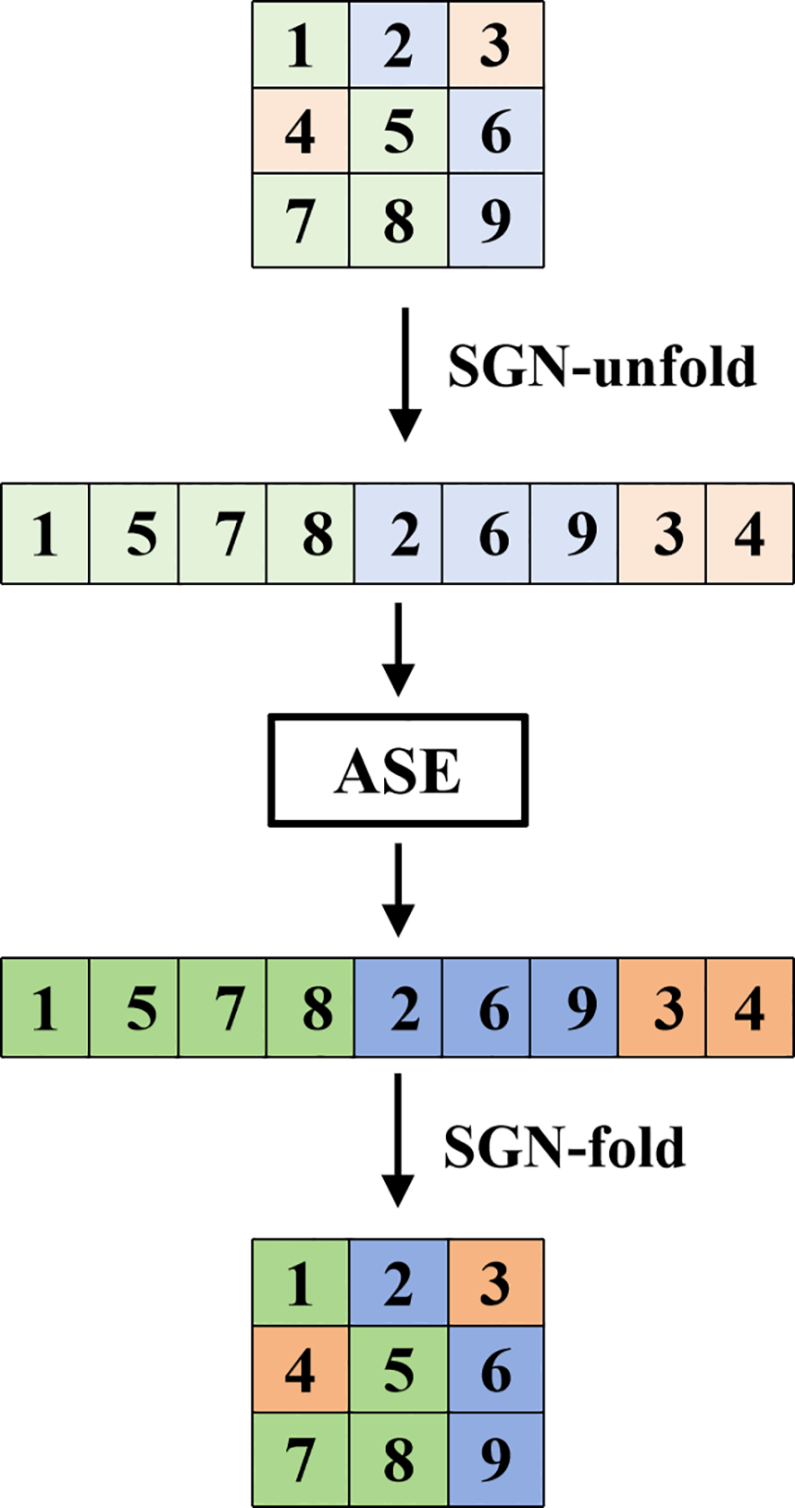
The function of SNG.

The ASSM, introduced within the neck network, serves as the core for constructing the efficient image processing unit, the ASSB. Early-stage PWD targets in UAV remote sensing imagery are characterized by sparse pixels, weak feature responses, and discontinuous spatial distributions. This necessitates an architecture with robust global relational modeling capabilities that transcend the limitations of localized receptive fields. By introducing the innovative Attentive State-Space Equation (ASE), the ASSM breaks the causal modeling limitations inherent to traditional Mamba models during sequence processing (Gu and Dao, 2023). It endows the model with an omni-directional, non-causal modeling capability similar to ViT (Dosovitskiy et al., 2020), providing a rigorous mathematical foundation for capturing sparse pathological features within complex forest backgrounds (Guo et al., 2024; Zhu et al., 2024). The detailed topological structure of the ASSM is illustrated in **Figure 10**. Within this module, the Scanning Network Generator (SNG) plays a pivotal role in multi-directional feature extraction; its specific operational mechanism and functional impact on spatial information encoding are further depicted in **Figure 11**.

During the discretization evolution of the system, the Attentive State-Space Equation (ASE) performs a critical extension based on the linear mapping of traditional State Space Models (SSMs). While the standard state-space equations are defined by (Equation 1), the ASE innovatively introduces an Instance-specific Prompt Matrix (P) to overcome the inherent deficiency where the C matrix can only perceive historical sequence information. The output equation is reconstructed as:

(5) $$\begin{array}{c} y_{i}=(C+P)h_{i}+Dx_{i} \end{array}$$

Where $h_{i}$ represents the hidden state, C is the standard output matrix, and D denotes the feed-forward term. By learning the feature sets of semantically similar pixels across the entire image, the Prompt Matrix ( P) functions as an “attentive prompt” that compensates for the information of unscanned pixels in the sequence. Through this residual addition mechanism, the consolidated matrix C+P acts similarly to the “Query” in attention mechanisms (Vaswani et al., 2017), enabling the model to transcend spatial distance constraints and precisely localize distant critical pixels.

Compared to the traditional Mamba architecture, which incurs substantial computational overhead by employing four-directional scanning to mitigate causality defects, the ASE based on (Equation 5) achieves global feature capture with just a single scan. This mechanism ensures the completeness of global perception while significantly reducing inference latency, allowing the model to more efficiently isolate subtle early-stage infection signals from the macro-canopy context.

Furthermore, the SNG mechanism integrated within the module further strengthens feature representation. Driven by semantic similarity, this mechanism guides deep interaction between pixels that are spatially distant but semantically related, effectively mitigating the signal attenuation problem during long-distance feature transmission. This design ensures that when processing small-scale targets such as early-stage infected pine trees, the model can fully leverage rich global semantic context for accurate localization and discrimination, thereby significantly improving detection precision in complex forest environments.

### 2.8 Prediction layer

**Figure 12:**
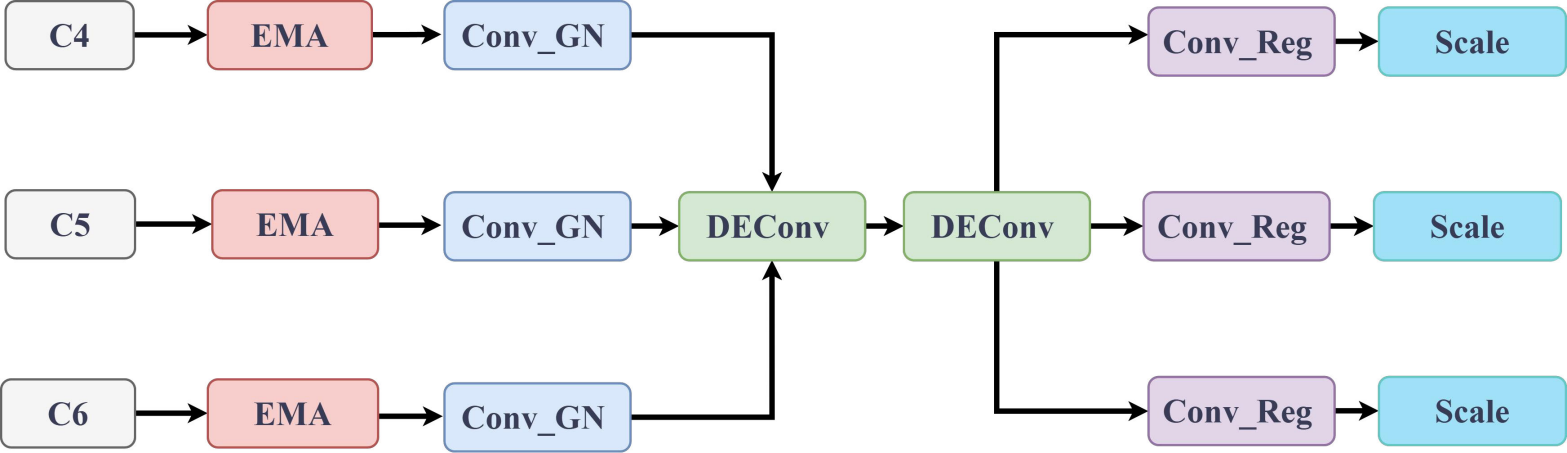
Structure of the LSDECD detection head.

**Figure 13:**
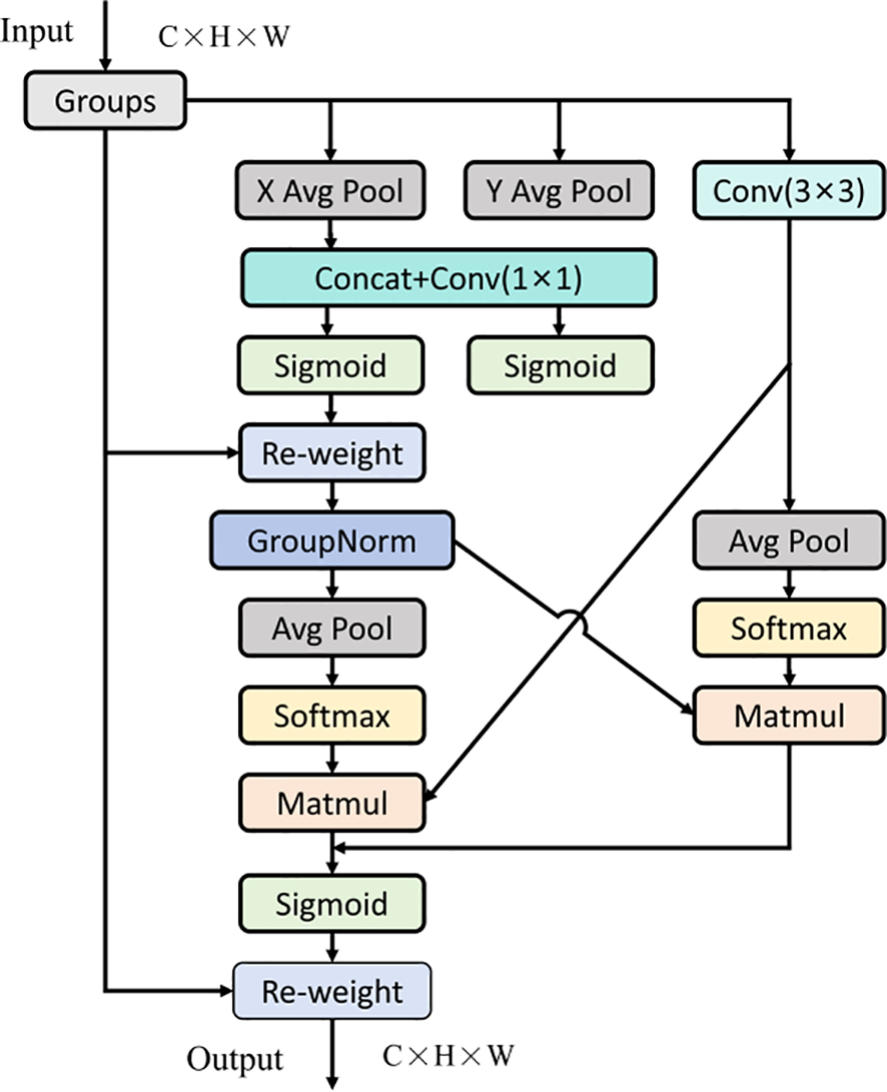
Structure of the EMA module.

The Prediction Layer is responsible for mapping the extracted high-level semantic features into final detection bounding boxes and category confidence scores. To address the fundamental pain points of extremely weak feature responses and high localization sensitivity inherent to early-stage PWD lesions in complex backgrounds (Liu et al., 2020), this study constructs an LSDECD architecture, whose topological structure is illustrated in **Figure 12**. The LSDECD leverages the DEConv operator to refine boundary localization by capturing subtle texture gradients. Furthermore, the model integrates the EMA mechanism (Ouyang et al., 2023), as depicted in **Figure 13**, to achieve high-precision capture and feature calibration of minute targets through cross-spatial channel interactions. This synergistic design of attention-guided feature recalibration and the detail-enhanced prediction head ensures robust detection performance in dense and cluttered forest environments.

Prior to the detection head, the EMA mechanism is introduced to recalibrate the fused features. A primary difficulty in early PWD detection is that complex “spectrally similar objects” (e.g., light and shadow flickers, dead vegetation) can easily submerge weak target signals. The EMA captures deep cross-spatial and cross-channel dependencies through parallelized dimensional encoding. Its core logic lies in utilizing multi-scale convolutional kernels to extract features, subsequently reallocating feature weights via global pooling and Softmax functions. This design adaptively suppresses background noise and significantly enhances the feature activation intensity in channels corresponding to small targets, ensuring a high SNR at the input stage of the detection head.

To guarantee localization precision, the LSDECD incorporates Re-parameterized DEConv. Since early-infected trees have a minimal pixel span, conventional convolutions struggle to accurately delineate their edge gradients. DEConv integrates differential convolution operators, which mathematically extract gradient difference information between neighboring pixels. This operation can be formulated as Equation 6:

(6) $$\begin{array}{c} y=w_{conv}*x+w_{diff}*\Delta x \end{array}$$

Where $w_{conv}$ is the convolutional kernel, x is the input sequence, $w_{diff}$ is the differential weight that determines the model’s sensitivity to “variations,” and Δx measures the rate of change (i.e., trends or abrupt mutations) in the signal between adjacent moments. Δx captures the subtle color transitions between lesions and the healthy canopy. During the training phase, the model learns intricate geometric details through a multi-branch architecture. In the inference phase, structural re-parameterization techniques (Ding et al., 2021) are employed to equivalently fuse these multi-branch weights into a single-path convolution. This “training-time augmentation, inference-time efficiency” strategy enables the detection head to significantly enhance the regression precision of minute lesion boundaries without incurring any additional computational overhead. Consequently, this effectively alleviates the common localization drift issue in small object detection.

Finally, through a shared-weight convolutional design, the LSDECD substantially compresses the parameter volume of the prediction head, facilitating efficient deployment on embedded UAV platforms (Fouda et al., 2022). In summary, the global perception of the EMA and the local detail enhancement of the LSDECD work synergistically to create a “perceptually acute, precisely localized, and inference-efficient” prediction framework. This architecture successfully resolves the challenge of low recognition rates for early PWD targets while comprehensively optimizing the model’s robustness to meet real-time field monitoring requirements.

### 2.9 ESE-PWDNet Design of the model loss function

To accommodate the diverse scenarios and high-precision requirements inherent in PWD detection, the ESE-PWDNet model employs a composite loss function that integrates classification loss, bounding box regression loss, and confidence loss (Jocher et al., 2021). Specifically, the Focaler-MPDIoU Loss—a synergistic fusion of Focaler-Intersection over Union (Focaler-IoU) (Zhang and Zhang, 2024) (which focuses on hard examples) and MPDIoU (Ma and Xu, 2023) (which ensures precise localization based on minimum point distance)—is developed to simultaneously optimize classification performance and localization accuracy in complex detection environments.

Focaler-IoU (Zhang and Zhang, 2024) is a bounding box regression loss function designed to enhance detector performance by adaptively prioritizing specific regression samples. Its core principle lies in reconstructing the standard IoU loss through linear interval mapping. This mechanism addresses the impact of imbalanced hard and easy sample distributions on bounding box regression—a critical factor frequently neglected in conventional IoU loss functions. By mathematically re-weighting these distributions, Focaler-IoU effectively compensates for the inherent deficiencies of existing regression methods.

The Focaler-IoU adjusts the loss based on the Intersection over Union (IoU) value, as defined in Equation 7:

(7) $$\begin{array}{c} loU^{focaler}=\{\begin{array}{cc} 0, & loU<d\\ \frac{loU-d}{u-d}, & d\leq loU\leq u\\ 1, & loU>u \end{array} \end{array}$$

Where d and u represent the lower and upper thresholds, respectively. By setting the loss to 0 when loU<d and to 1 when loU>u, the function becomes sensitive only within the d≤loU≤u interval. This allows the model to concentrate on samples with moderate overlap between the predicted and ground-truth boxes—targets that are neither too easy nor too difficult—thereby facilitating more effective feature extraction from moderately challenging samples.

**Figure 14:**
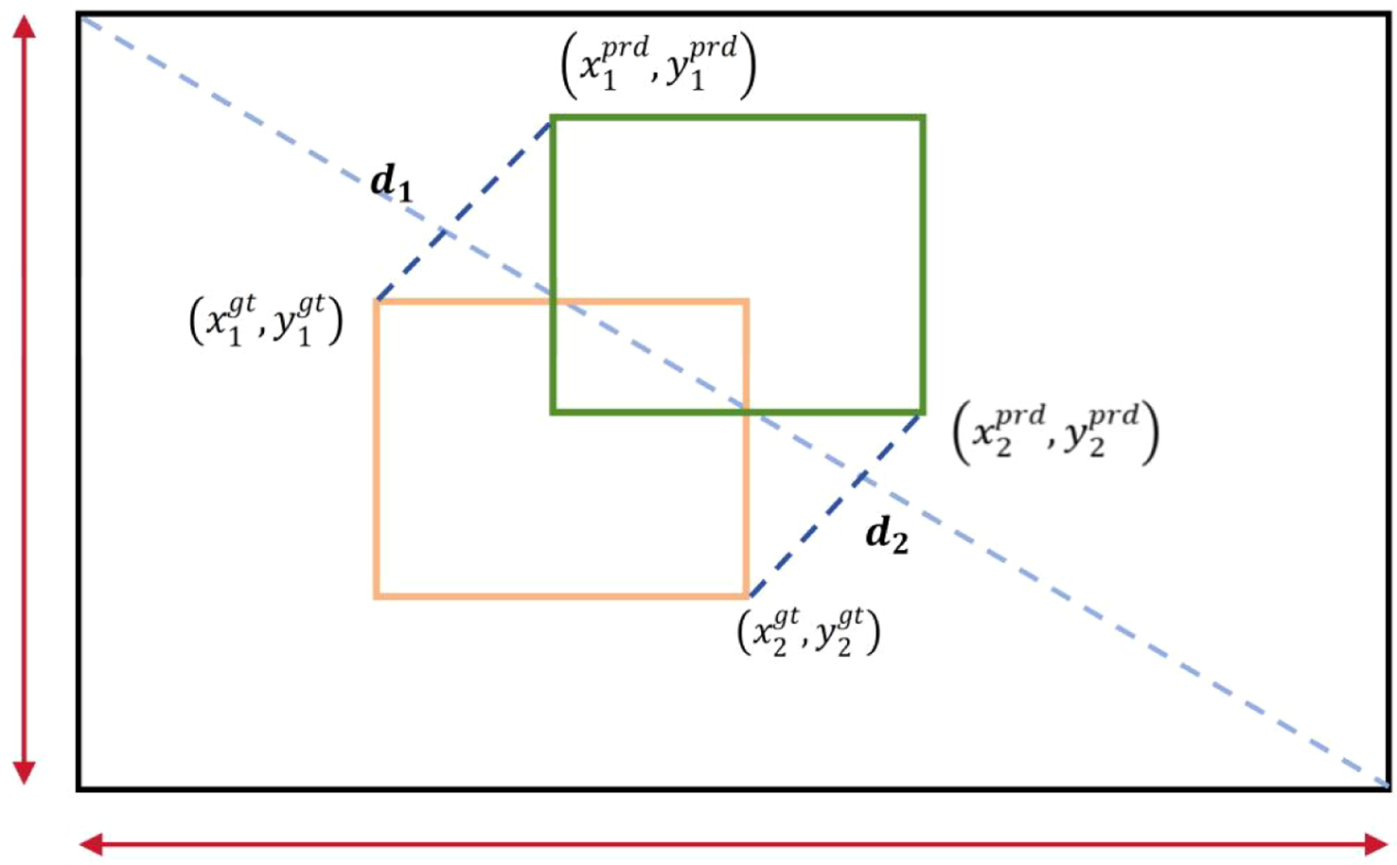
Geometric elements of the MPDIoU loss.

In the domain of object detection, Minimum Point Distance Intersection over Union (MPDIoU) (Ma and Xu, 2023)is an efficient bounding box regression loss function proposed to overcome the limitations of traditional IoU variants (e.g., GIoU, DIoU, CIoU) when dealing with varying aspect ratios, extreme scales, and complex overlapping scenarios. While conventional functions focus primarily on area overlap, center-point distance, or aspect ratio, MPDIoU optimizes the geometric distance by directly minimizing the distance between the top-left and bottom-right corners of the predicted and ground-truth boxes. This is predicated on a fundamental geometric theorem: two axis-aligned rectangles are identical in shape, position, and size if and only if their top-left and bottom-right coordinates perfectly coincide (**Figure 14**).

The MPDIoU formula is defined as:

(8) $$\begin{array}{c} \left\{ \begin{array}{c} d_{1}^{2}={\left(x_{1}^{prd}-x_{1}^{gt}\right)}^{2}+{\left(y_{1}^{prd}-y_{1}^{gt}\right)}^{2}\\ d_{2}^{2}={\left(x_{2}^{prd}-x_{2}^{gt}\right)}^{2}+{\left(y_{2}^{prd}-y_{2}^{gt}\right)}^{2}\\ MPDIoU=IoU-\frac{d_{1}^{2}}{w^{2}+h^{2}}-\frac{d_{2}^{2}}{w^{2}+h^{2}} \end{array}\right. \end{array}$$

The formula first computes the squared Euclidean distance $d_{1}^{2}$ between the predicted top-left coordinates ( $x_{1}^{prd}$, $y_{1}^{prd}$) and the ground-truth top-left coordinates ( $x_{1}^{gt}$, $y_{1}^{gt}$), as well as the squared Euclidean distance $d_{2}^{2}$ between the predicted bottom-right coordinates ( $x_{2}^{prd}$, $y_{2}^{prd}$) and the ground-truth bottom-right coordinates ( $x_{2}^{gt}$, $y_{2}^{gt}$); these two terms directly reflect the absolute positional shift of the predicted box in 2D space. Subsequently, MPDIoU scales these two distance penalties by the normalization factor $w^{2}+h^{2}$, which is the sum of squares of the input image box width *w* and height *h*,ensuring that the magnitude of the penalty terms is comparable to the IoU itself (with a value range of [0,1]), while also endowing the loss function with scale invariance to prevent gradient contribution imbalance between large and small boxes caused by target size differences. Ultimately, the complete expression of MPDIoU is (Equation 8), where the IoU term drives the predicted box to achieve large-area overlap with the ground-truth box, while the two normalized distance penalty terms force the model’s predicted corners to converge toward the ground-truth corners.Where *w* and *h* are the width and height of the input image. The corresponding loss function is defined as Equation 9:

(9) $$\begin{array}{c} L_{MPDIoU}=1-MPDIoU \end{array}$$

The integration of Focaler and MPDIoU forms a logical complementarity: the Focaler mechanism serves as a “filter” to identify and focus on the most valuable lesion samples among numerous candidates, while MPDIoU provides “fine-grained alignment” for high-precision geometric calibration of those selected samples. This “focus-then-refine” strategy enables ESE-PWDNet to effectively mitigate poor localization precision and high false-positive rates in early-stage PWD detection without increasing computational complexity, providing a solid mathematical foundation for robust monitoring in complex forest environments.

## 3 Experimental results

### 3.1 Experimental details

The hardware and software configurations, model training parameters, and experimental environment are as follows: the hardware setup includes an NVIDIA RTX 4060 GPU and an AMD 7940HX processor. The software environment is based on Python 3.9 and PyTorch 2.4.1, utilizing CUDA 12.6 for GPU acceleration. During model training, the initial learning rate was set to 0.001, the SGD optimizer was employed, and the batch size was set to 8. To ensure the reliability and comparability of the results, all models were trained and validated on the same Pine Wilt Disease (PWD) dataset.

### 3.2 Comparison of experimental results and analysis

To comprehensively benchmark the performance superiority of the proposed ESE-PWDNet—particularly its efficacy in early-stage PWD detection—this study conducted rigorous comparative evaluations against several mainstream object detection architectures. The selected baselines encompass the classic two-stage detector, Fast R-CNN (Girshick, 2015), as well as prominent lightweight one-stage detectors, including YOLOv5n (Jocher et al., 2021), YOLOv8n (Jocher et al., 2023), and ShuffleNet V2 (Ma et al., 2018).

As detailed in **Table 2**, the quantitative comparison clearly substantiates that ESE-PWDNet exhibits superior, state-of-the-art detection capabilities when addressing the profound challenges of pest and disease identification in their nascent stages.

**Table 2:** Performance comparison among different lightweight models.

Model	Stage	Evaluation indicators					
		P(%)	R(%)	mAP50(%)	GFLOPs(G)	Parameters(M)	FPS
Fast-RCNN(R50-FPN)	PWD-pre	56.3	66.2	56.3	134.17	41.8	41.1
	PWD-advanced	64.8	74.0	64.0			
Hyper-YOLO	PWD-pre	74.7	64.8	71.6	9.5	3.62	174
	PWD-advanced	77.4	79.9	81.6			
YOLOv8n	PWD-pre	71.6	74.5	73.4	6.8	2.68	84.2
	PWD-advanced	75.4	81.4	79.7			
YOLOv11n	PWD-pre	71.1	67.2	70.0	6.5	2.62	210
	PWD-advanced	76.6	77.7	81.0			
YOLOv12n	PWD-pre	71.0	63.2	60.1	**5.8**	**2.51**	**162.5**
	PWD-advanced	75.4	77.7	76.5			
RT-DETR-R18	PWD-pre	67.1	66.1	62.8	57	19.88	82.9
	PWD-advanced	76.6	**82.9**	82.5			
ESE-PWDNet	PWD-pre	**75.9**	**75.1**	**73.5**	6.5	2.70	124
	PWD-advanced	78.6	81.2	**82.9**			

Bold values indicate the best results.

Based on the quantitative evaluation presented in **Table 2**, several critical analytical conclusions can be drawn regarding the performance of the proposed ESE-PWDNet:

Detection Accuracy: The ESE-PWDNet demonstrates superior sensitivity to PWD lesions when compared against established baseline models. In the advanced infection stage (*PWD-advanced*), its Precision (P) and mAP50 reached 78.6% and 82.9%, respectively, establishing the highest performance levels among all evaluated networks. In the critical early infection stage (*PWD-pre*), its Precision peaked at 75.9% with an mAP50 of 73.5%. While YOLOv8n (Jocher et al., 2023) achieved a closely trailing mAP50 (73.4%) in the *PWD-pre* stage, its Precision was significantly lower (71.6%). Notably, despite being the latest iterations in the YOLO family, YOLOv11n (Khanam and Hussain, 2024) and YOLOv12n (Sapkota and Karkee, 2025) exhibited severe performance degradation when detecting microscopic early-stage targets, with YOLOv12n’s mAP50 plummeting to 60.1%. Furthermore, although the recent Hyper-YOLO (Feng et al., 2024) demonstrated competitive Precision (74.7%), its mAP50 (71.6%) remained inferior to the proposed model. These results indicate that state-of-the-art (SOTA) general-purpose models struggle to capture the weak semantic features of nascent lesions, whereas the ESE-PWDNet possesses highly specialized target perception capabilities.

Recall Rate: In the most challenging *PWD-pre* stage, the ESE-PWDNet achieved the highest Recall (R) of 75.1%, substantially outperforming YOLOv8n (74.5%) and drastically surpassing the newer YOLOv11n (67.2%) and YOLOv12n (63.2%). This metric translates to a minimized missed detection rate (i.e., false negative rate), which is paramount for truncating the disease transmission chain through early intervention. In the *PWD-advanced* stage, the ESE-PWDNet recorded a strong Recall of 81.2%. While the heavy-weight RT-DETR-R18 (Zhao et al., 2024) marginally edged out with the highest Recall (82.9%) in the advanced stage, its early-stage Recall was highly inadequate (66.1%). Evaluated in conjunction with its superior Precision, the ESE-PWDNet offers the most robust and optimally balanced detection performance across the entire infection lifecycle.

Computational Complexity and Efficiency: The ESE-PWDNet requires only 6.5 GFLOPs of computational overhead and 2.70M parameters, sharing an identical computational footprint with YOLOv11n but delivering vastly superior early-stage accuracy. While YOLOv12n (5.8 GFLOPs) offers a slightly lower computational cost, its unacceptable drop in early infection detection precision renders it unsuitable for precise forest pest control. Conversely, Fast R-CNN (Girshick, 2015) (134.17 GFLOPs) and the Transformer-based RT-DETR-R18 (57 GFLOPs, 19.88M parameters) impose massive computational burdens that far exceed the limits of UAV-mounted edge devices. Furthermore, the ESE-PWDNet achieves an impressive inference speed of 124 FPS. Although Hyper-YOLO and YOLOv11n present higher frame rates, 124 FPS is more than sufficient to guarantee real-time, low-latency target tracking and dynamic warning during UAV flight patrols.

Comprehensive Analysis: A holistic evaluation reveals that the ESE-PWDNet decisively overcomes the inherent limitations of recent SOTA general-purpose detectors in identifying weak, early-stage PWD signals. By maintaining the highest Precision and a remarkably low missed detection rate (highest R-value in the *PWD-pre* stage), the model significantly outperforms all baselines where it is most critical. Concurrently, by constraining the computational complexity to 6.5 GFLOPs and securing a real-time inference speed of 124 FPS, the ESE-PWDNet successfully avoids the computational redundancy of large-scale models like RT-DETR-R18, while delivering far superior detection accuracy compared to ultra-lightweight architectures like YOLOv12n. This underscores its high feature extraction efficiency and immense practical viability for embedded forestry deployments.

**Figure 15:**
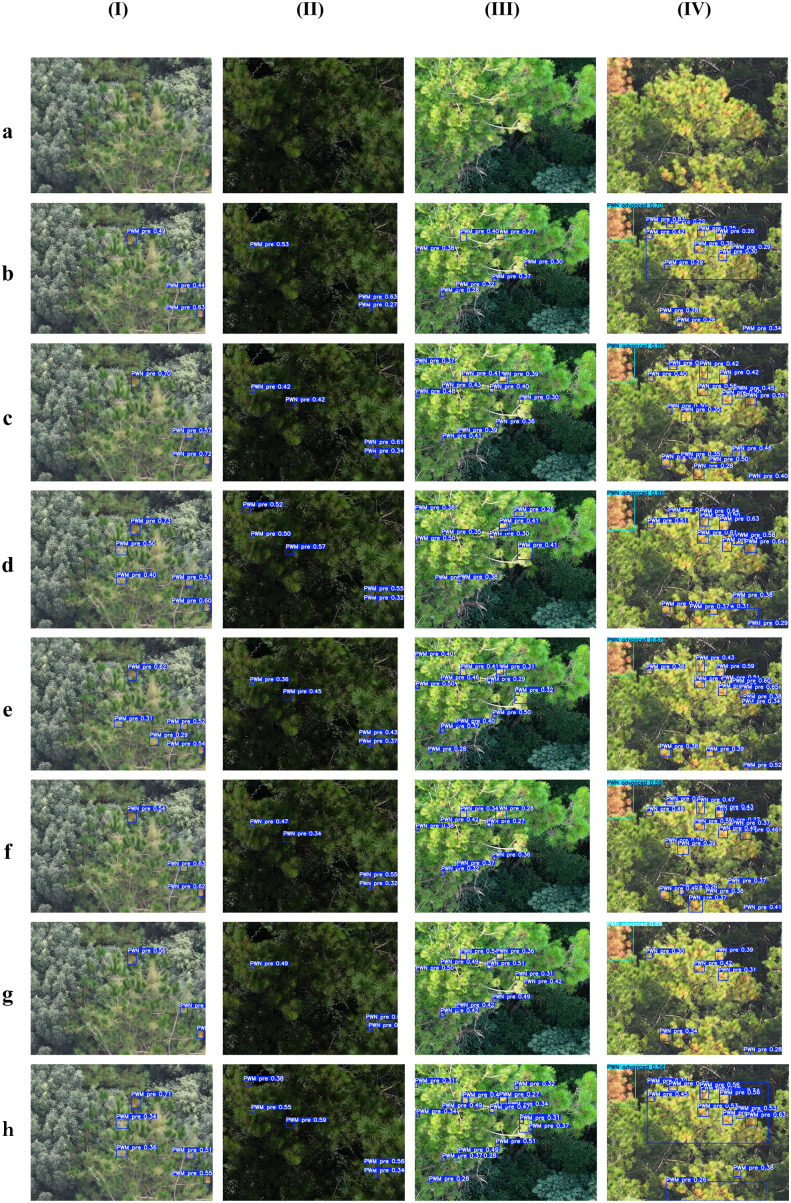
Detection effects of different models on the target PWD dataset. **(a)** Original image, **(b)** Fast-RCNN(R50-FPN), **(c)** Hyper-YOLO, **(d)** YOLOv8n, **(e)** YOLOv11n, **(f)** YOLOv12n, **(g)** RT-DETR-R18, **(h)** ESE-PWDNet.

To qualitatively benchmark the performance of the evaluated models, four representative high-resolution images were selected from the test dataset for visual analysis. The visualization of these detection results is presented in **Figure 15**. These selected samples deliberately encompass distinct infection stages (*PWD-pre* vs. *PWD-advanced*), various diurnal periods (morning, noon, and evening), and diverse UAV acquisition angles (nadir vs. oblique), thereby providing a rigorous and holistic assessment of each model’s real-world detection robustness.

To qualitatively evaluate the detection efficacy of the proposed ESE-PWDNet, **Figure 15** presents a visual comparison of detection results across several SOTA architectures. In complex forest environments where early-stage *PWD-pre* targets occupy only a limited number of pixels, the baseline models exhibit varying degrees of performance degradation. Notably, the most recent iterations in the YOLO family, YOLOv11n and YOLOv12n, suffer from severe missed detections (false negatives), as evidenced in **Figures 15e, f**. These general-purpose detectors struggle to distinguish the subtle chlorosis and faint yellowing of pine needles from the cluttered healthy canopy background. Similarly, the Transformer-based RT-DETR-R18 (**Figure 15g**) fails to localize several minute lesion targets amidst dense vegetation and severe shadow interference, indicating a limitation in capturing sparse pathological features.

In contrast, the ESE-PWDNet (**Figure 15h**) achieves the most robust and accurate detection performance across all test scenarios. It successfully identifies all pathological targets, including those characterized by extreme small-scale and low-contrast features that were overlooked by the SOTA baselines. The predicted bounding boxes generated by ESE-PWDNet are remarkably tight and precise, which can be attributed to the enhanced geometric constraints provided by the composite Focaler-MPDIoU loss and the refined localization capability of the LSDECD head. Furthermore, the high confidence scores associated with the ESE-PWDNet’s predictions demonstrate the effectiveness of the HSM-SSD and EMA modules in amplifying weak pathological signals while suppressing complex environmental noise. These visual results intuitively confirm that ESE-PWDNet significantly reduces the missed detection rate in early-stage PWD monitoring, providing a highly reliable technical foundation for real-time UAV forest health assessments in resource-constrained environments.

### 3.3 Results and analysis of ablation experiments

To verify the incremental contributions of the key modules in ESE-PWDNet to early-stage PWD small-target detection, several sets of ablation experiments were conducted. The performance was analyzed across five dimensions: mAP, GFLOPs, Parameters, Precision (P), and Recall (R). The experiments sequentially evaluated the contributions of the ASSB (in the feature fusion layer), EMA, LSDECD, and Focaler-MPDIoU loss function. Model 1 serves as the baseline, utilizing only the EF-Backbone without any of the aforementioned modules. The quantitative results of the ablation study are summarized in **Table 3**.

**Table 3:** Results of the ablation experiments of the ESE-PWDNet model.

Model	ASSB	LSDECD	EMA	Focaler-MPDIoU	Stage	Evaluation indicators				
						P	R	mAP50	GFLOPs	Parameters
						(%)	(%)	(%)		(M)
1					PWD-pre	73.9	72.1	71.6	7.2	2.6
2	✓				PWD-pre	74.0	75.2	73.2	7.1	2.7
3	✓	✓			PWD-pre	75.4	72.8	73.1	6.6	2.6
4	✓	✓	✓		PWD-pre	74.6	76.1	72.5	6.6	2.6
5	✓	✓	✓	✓	PWD-pre	75.9	75.1	73.5	6.6	2.6

Based on the quantitative data presented in **Table 3**, the following analytical conclusions can be drawn:

Impact of the ASSB: The integration of the ASSB concurrently enhances feature capture and optimizes computational efficiency. Upon its addition (Model 2), the computational overhead decreased from 7.2 GFLOPs to 7.1 GFLOPs. Despite a marginal 0.1M parameter increase, this structure significantly heightened the model’s sensitivity to early-stage lesions, boosting Recall from 72.1% to 75.2% and mAP50 by 1.6% (from 71.6% to 73.2%). This indicates that by optimizing the hierarchical feature extraction architecture, the ASSB effectively strengthens the perceptual capacity of the lightweight model for *PWD-pre* targets.

Efficiency of the LSDECD: The LSDECD substantially reduces computational complexity and storage requirements by mathematically refining the feature representation paths. With the introduction of the LSDECD (Model 3), GFLOPs dropped significantly from 7.1 to 6.6, and the parameter count reverted to its highly efficient baseline of 2.6M. Concurrently, while achieving model lightweighting, this architecture enhanced feature discriminability, raising early-stage Precision from 74.0% to 75.4%. Despite slight fluctuations in Recall, the LSDECD achieves an exceptional trade-off between scale optimization and high precision, rendering it ideal for resource-constrained edge computing deployments.

Effectiveness of the EMA: The EMA mechanism significantly improves the capture accuracy for minute, early-stage targets by enhancing cross-spatial and cross-channel interactions (Ouyang et al., 2023), all while maintaining strictly constant computational costs (6.6 GFLOPs, 2.6M parameters). After incorporating the EMA (Model 4), the early-stage Recall surged from 72.8% to 76.1%, drastically reducing the rate of missed detections. This empirical evidence proves that the EMA effectively mitigates complex forest background interference by selectively focusing on critical feature regions without exacerbating the inference burden.

Optimization via Focaler-MPDIoU: The composite Focaler-MPDIoU loss function (Ma and Xu, 2023; Zhang and Zhang, 2024) further refines bounding box regression, specifically improving the geometric localization for early-stage small targets. With zero additional computational overhead (Model 5), Precision increased to 75.9%, and the mAP50 reached a peak of 73.5%. This demonstrates that Focaler-MPDIoU, through its rigorous geometric constraints and adaptive sample focusing, enables optimal feature representation and detection performance in the critical *PWD-pre* stage.

**Figure 16:**
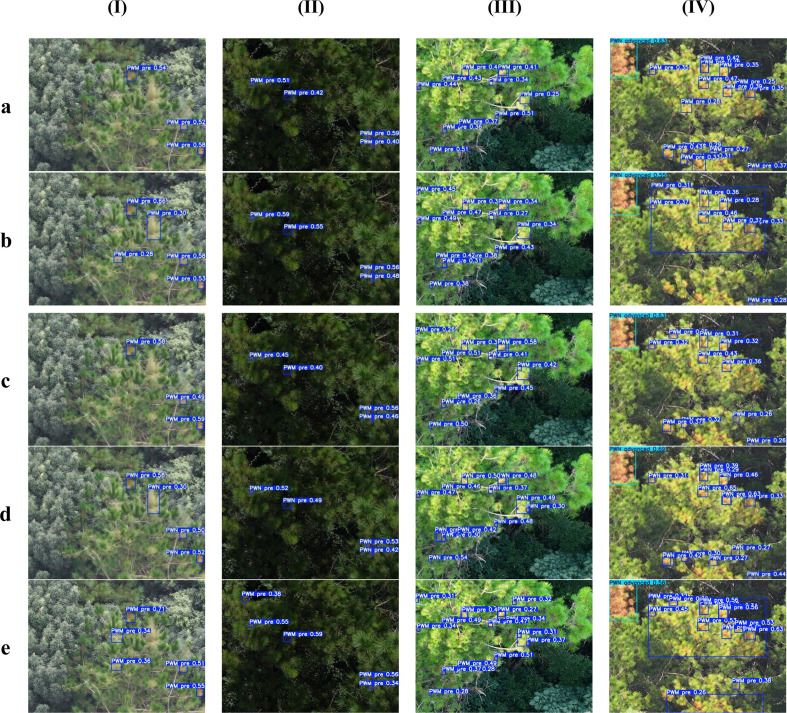
Comparison chart of ablation experiments **(a)** EF-Backbone, **(b)** EF-Backbone+ASSB, **(c)** EF-Backbone+ASSB+LSDECD, **(d)** EF-Backbone+ASSB+ LSDECD+EMA, **(e)** EF-Backbone+ASSB+ LSDECD+EMA+Focaler-MPDIoU.

The qualitative visual comparisons elucidating the incremental improvements of these ablation experiments are comprehensively depicted in **Figure 16**.

The visual comparison of the detection results (as shown in **Figure 16**) provides a clear trajectory of the incremental improvements offered by each module. The baseline EF-Backbone (a) exhibits a noticeable perceptual deficiency when dealing with complex backgrounds, particularly in samples (I) and (III), where missed detections occur for extremely small lesion areas with subtle color variations.

Following the introduction of the ASSB module (b), the model’s capability to capture early-stage sparse pathological features is significantly bolstered, successfully retrieving several previously lost small targets in the dark background of sample (II) and the densely distributed regions of sample (IV). Subsequently, the integration of the LSDECD module (c) further refines the quality of the bounding boxes; while maintaining a high recall rate, the delimited areas become more precise, effectively reducing interference from redundant background features. Through the synergistic optimization of the EMA mechanism and the Focaler-MPDIoU loss function (d, e), the final model demonstrates exceptional detection robustness.

Observations of samples (III) and (IV) reveal that the full version of the model not only accurately detects all minute lesions but also significantly increases the confidence scores of the detection boxes, with localization closely aligning with the actual discolored areas of the tree crowns. Overall, the visualization results intuitively prove that by integrating global contextual awareness and multi-scale attention mechanisms, ESE-PWDNet successfully overcomes the interference of complex forest backgrounds on small-target detection, achieving comprehensive and precise coverage of pathological targets across all infection stages.

In summary, the ESE-PWDNet model designed in this study utilizes various lightweight architectures and feature extraction enhancement strategies to improve the detection capability of small-scale PWD targets while ensuring real-time performance. It is highly suitable for resource-constrained edge computing environments and provides an efficient and reliable solution for the early monitoring of Pine Wilt Disease.

### 3.4 Robustness and generalization analysis

To systematically evaluate the reliability and practical deployment viability of the ESE-PWDNet in complex field environments, we conducted a series of targeted stress tests. These experiments meticulously scrutinized the model’s stability across varying stand structures, image acquisition parameters, and severe background interference (Woo et al., 2018).

Resilience to Forest Heterogeneity: We evaluated the model in both pure pine stands (homogeneous) and mixed forests (heterogeneous). Mixed forests inherently introduce significant spectral noise and textural interference due to the interleaved distribution of diverse tree species. Qualitative results demonstrate that the ESE-PWDNet maintains high identification consistency across both scenarios. Benefiting from the global semantic capture capabilities of the ASSM, the model effectively distinguishes early PWD signals from “spectrally similar objects” (e.g., senescent broadleaf foliage) (Syifa et al., 2020), showcasing its profound resilience to forest heterogeneity.

Adaptability to Varying UAV Altitudes: To verify the model’s adaptability to fluctuating UAV operational altitudes, we compared detection performance across different spatial resolutions. As flight altitude increases, the pixel occupancy of lesion targets diminishes drastically, exacerbating the small-target detection bottleneck (Liu et al., 2020). The empirical results demonstrate that the ESE-PWDNet, empowered by the detail enhancement mechanisms of the EFVLU for minute targets, maintains stable localization precision across various altitudes without experiencing significant performance degradation.

### 3.5 Comparison of different attention mechanisms

To verify the specific contribution and suitability of the EMA mechanism within the ESE-PWDNet architecture, this study conducted a benchmarking experiment against four representative attention mechanisms. The quantitative results are detailed in **Table 4**. The experimental data indicate that the EMA demonstrates a significant competitive advantage across the following dimensions:

**Table 4:** Comparison of different attention mechanisms.

Model	P(%)	R(%)	mAP@0.5(%)	Parameters(M)	GFLOPs
X+SimAM	73.3	75.0	72.4	2.7	6.4
X+CAFM	72.0	76.0	73.4	3.2	7.1
X+EfficientChannelAttention	73.9	75.2	73.3	2.7	6.4
X+EMA	75.9	73.1	73.5	2.6	6.5

Accuracy Dimension: The mAP50 of the EMA-equipped model reached 73.5%, surpassing established mechanisms such as SimAM (Yang et al., 2021). This substantiates the superiority of cross-dimensional interaction for capturing minute lesion features under the low-SNR conditions typical of complex forest environments.

Efficiency Dimension: The EMA exhibits exceptional lightweight characteristics. Its parameter volume (2.6M) and computational cost (6.5 GFLOPs) are the lowest among the compared schemes. Specifically, it reduced parameter redundancy by approximately 18.7% compared to the Coordinate Attention-based Fusion Module (CAFM) while achieving superior precision.

This “high-gain, low-consumption” profile confirms that the EMA can effectively suppress environmental noise through multi-scale parallel encoding without increasing on-device inference pressure. Consequently, it represents the optimal component choice for enhancing the perceptual recognition capability of early-stage PWD monitoring models.

## 4 Discussion

PWD is characterized by rapid transmission and a high fatality rate, with infected trees often progressing from initial symptoms to death within only a few dozen days. Therefore, early, accurate, and cost-effective monitoring methods are critical for containing the epidemic. Traditional ground survey methods are inefficient and struggle to meet the demands of large-scale real-time monitoring, while satellite remote sensing faces challenges in effectively detecting small-scale PWD targets. In recent years, with the advancement of UAV remote sensing and object detection algorithms, researchers have increasingly applied deep learning technologies to the automated identification of diseased trees. Specifically, lightweight small-target detection methods have become a pivotal direction for achieving precise PWD monitoring. Consequently, the ESE-PWDNet proposed in this study demonstrates superior identification accuracy in the early monitoring of PWD.

**Figure 17:**
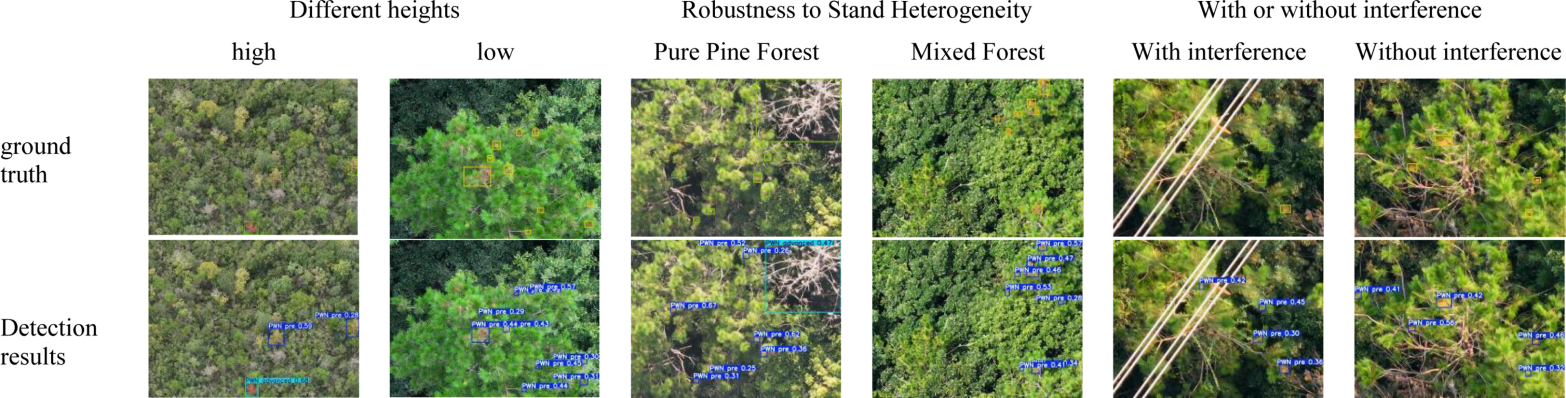
Experimental results of robustness and generalization.

Furthermore, validation across datasets encompassing multi-temporal, multi-view, and diverse heterogeneous forest backgrounds confirms that ESE-PWDNet possesses exceptional cross-regional generalization capabilities (**Figure 17**). In practical monitoring, significant differences exist between northern and southern pine forests in China regarding tree species composition (e.g., *Pinus massoniana* in the south vs. Pinus tabuliformis and *Pinus koraiensis* in the north) and site conditions. Even when the model was migrated to Xinbin Manchu Autonomous County, Fushun City, Liaoning Province—a region with vast differences in geographical environment and climate—ESE-PWDNet exhibited remarkable adaptability. Facing the unique phenological variations and complex mountainous terrain of northern forest areas, the model effectively overcame spectral feature fluctuations caused by cross-climatic backgrounds. This success is attributed to the integrated Attentive State Space Module (ASSB) for precise global semantic capture and the DEConv for sensitivity to subtle lesion edge gradients. Experimental results indicate that the model remains robust not only in southern forest areas like Nanjing but also in typical northern epidemic areas like Xinbin, Liaoning. This stable performance across large latitudinal gradients validates the universality of the model’s key pathological feature extraction for early PWD and provides a scientific and reliable algorithmic foundation for the dynamic monitoring and rapid early warning of pine forest epidemics across different eco-geographical zones nationwide.

The proposed ESE-PWDNet aims to significantly reduce computational complexity while enhancing the capture capability for PWD small targets in complex forest environments. In the backbone, the EFVLU was designed based on the CSPNet framework. Leveraging the efficiency of the Efficient ViM block and integrating a CGLU at the tail, this design enables the model to capture long-range global dependencies while maintaining extremely low computational overhead. Simultaneously, the soft-attention mechanism introduced by the CGLU enhances local modeling, allowing for more precise extraction of small-target details. In the feature fusion layer, a novel neck network was designed by combining the Attention State Space Block (ASSB) with the PANet architecture. The ASSB ensures high efficiency even when processing high-resolution imagery, while the PANet further deepens the perception of contextual information through bidirectional multi-scale feature aggregation, significantly improving detection robustness for multi-scale targets. Finally, the prediction layer incorporates the Efficient Multi-scale Attention (EMA) and the Lightweight Shared Detail Enhanced Convolutional Detection Head (LSDECD). This configuration optimizes perceptual sensitivity and localization precision for small targets with almost no increase in inference cost. Comparative analysis shows that ESE-PWDNet achieves an optimal balance between detection accuracy and inference speed, demonstrating immense potential for practical applications such as embedded edge devices.

Beyond algorithmic superiority, the practical promotion of automated PWD monitoring by forestry authorities hinges strictly on economic feasibility and operational simplicity. Regarding data collection costs and operational difficulty, this study demonstrates that high-precision early PWD detection can be achieved using affordable, consumer-grade UAVs (e.g., the DJI Air series) equipped with standard RGB cameras, entirely circumventing the need for exorbitant multispectral or hyperspectral sensors. The integration of automated flight planning and obstacle avoidance in modern consumer UAVs drastically lowers the operational threshold, allowing frontline forestry personnel to execute standardized aerial surveys with minimal specialized training. In terms of computing costs, the ultra-lightweight architecture of ESE-PWDNet (requiring only 2.70M parameters and 6.5 GFLOPs) fundamentally eliminates the reliance on expensive cloud-based GPU servers or heavy ground control stations. The model can be seamlessly deployed on low-cost, low-power edge computing boards (such as the NVIDIA Jetson series) directly mounted on the UAV, or run locally on standard field laptops. This paradigm shifts the processing pipeline from “offline cloud computing” to “real-time edge inference,” providing forestry management departments with a highly scalable, low-cost, and easily deployable technical solution for large-scale, dynamic forest health monitoring.

## 5 Conclusion

To meet the requirements for real-time detection in resource-constrained forest environments and to enhance the detection of small-scale PWD lesion areas, this study proposes the lightweight detection model ESE-PWDNet. Experimental results demonstrate that ESE-PWDNet achieves exceptional detection accuracy while maintaining low computational complexity (6.5 GFLOPs and parameters of only 2.6M). Specifically, for the most challenging early infection stage (*PWD-pre*), the model achieved a Precision (P) of 75.9%, a Recall (R) of 75.1%, and an mAP50 of 73.5%. In the advanced stage (*PWD-advanced*), performance further improved, with mAP50 reaching 82.9%, and Precision and Recall reaching 78.6% and 81.2%, respectively.

In summary, the model effectively balances scale and efficiency while significantly strengthening the perception of early-stage small targets. It provides a highly efficient and reliable lightweight solution for PWD prevention and control in resource-constrained scenarios such as UAV inspections. Nevertheless, the early identification of PWD remains a challenge. Future research will focus on further improving the model’s detection capabilities for deployment on UAV platforms, enabling the timely and accurate identification of infected trees to facilitate prompt intervention and treatment.

## Data Availability

The original contributions presented in the study are included in the article/supplementary material. Further inquiries can be directed to the corresponding authors.
